# Understanding how high stocking densities and concurrent limited oxygen availability drive social cohesion and adaptive features in regulatory growth, antioxidant defense and lipid metabolism in farmed gilthead sea bream (*Sparus aurata*)

**DOI:** 10.3389/fphys.2023.1272267

**Published:** 2023-10-04

**Authors:** Paul G. Holhorea, Fernando Naya-Català, Álvaro Belenguer, Josep A. Calduch-Giner, Jaume Pérez-Sánchez

**Affiliations:** Nutrigenomics and Fish Growth Endocrinology Group, Institute of Aquaculture Torre de la Sal (IATS, Spanish National Research Council (CSIC)), Castellón, Spain

**Keywords:** gilthead sea bream, stocking density, oxygen availability, welfare indicators, antioxidant defense, lipid metabolism, energy sensing, growth regulation

## Abstract

The study combined the use of biometric, behavioral, physiological and external tissue damage scoring systems to better understand how high stocking densities drive schooling behavior and other adaptive features during the finishing growing phase of farmed gilthead sea bream in the Western Mediterranean. Fish were grown at three different final stocking densities (LD, 8.5 kg/m^3^; MD, 17 kg/m^3^; HD, 25 kg/m^3^). Water oxygen concentration varied between 5 and 6 ppm in LD fish to 3–4 ppm in HD fish with the summer rise of water temperature from 19°C to 26°C (May–July). HD fish showed a reduction of feed intake and growth rates, but they also showed a reinforced social cohesion with a well-defined endogenous swimming activity rhythm with feeding time as a main synchronization factor. The monitored decrease of the breathing/swimming activity ratio by means of the AEFishBIT data-logger also indicated a decreased energy partitioning for growth in the HD environment with a limited oxygen availability. Plasma glucose and cortisol levels increased with the rise of stocking density, and the close association of glycaemia with the expression level of antioxidant enzymes (*mn-sod, gpx4, prdx5*) in liver and molecular chaperones (*grp170, grp75*) in skeletal muscle highlighted the involvement of glucose in redox processes via rerouting in the pentose-phosphate-pathway. Other adaptive features included the depletion of oxidative metabolism that favored lipid storage rather than fatty acid oxidation to decrease the oxygen demand as last electron acceptor in the mitochondrial respiratory chain. This was coincident with the metabolic readjustment of the Gh/Igf endocrine-growth cascade that promoted the regulation of muscle growth at the local level rather than a systemic action via the liver Gh/Igf axis. Moreover, correlation analyses within HD fish displayed negative correlations of hepatic transcripts of *igf1* and *igf2* with the data-logger measurements of activity and respiration, whereas the opposite was found for muscle *igf2, ghr1* and *ghr2*. This was indicative of a growth-regulatory transition that supported a proactive instead of a reactive behavior in HD fish, which was considered adaptive to preserve an active and synchronized feeding behavior with a minimized risk of oxidative stress and epidermal skin damage.

## 1 Introduction

Global aquaculture production of aquatic animals increased at an average rate of 2.2% from 1990 to 2020 until reaching a milestone of 88 million tonnes per year (FAO, 2022). This increase of animal aquaculture production mostly supported the augmented human *per capita* consumption of fish from 14 kg (live weigh equivalent) in 1990 to 20.2 kg in 2020. However, the intensification of aquaculture production must deal with inadequate stocking densities that may increase the risk of health issues and welfare impairments due to feed competition and aggressive interactions among other stressful events (Ellis et al., 2002; North et al., 2006; Baldwin, 2011; Jia et al., 2016; Liu et al., 2017; Wu et al., 2018). The legislation limiting stocking density can contribute to support the expansion of a more sustainable and ethical aquaculture production, but a more rational approach might be to define acceptable levels of different welfare indicators for each particular species, life-stage and production system (van de Vis et al., 2020; Saraiva et al., 2022). Certainly, important research efforts have been made over the last decade for finding the golden stocking density of farmed fish, combining criteria of economic profitability with the increasing pressure of consumers in developed countries for an enhanced control and regulation in welfare assurance schemes (Noble et al., 2018). In land-based systems, negative effects on feed utilization and physiological stress markers were found in Atlantic salmon (*Salmo salar*) at stocking densities above 75 kg/m^3^, but densities above 25 kg/m^3^ reduced feed intake and growth of adult fish kept in sea cages (Calabrese et al., 2017). Signs of external tissue lesions, and impaired growth and feed utilization also occurred above 20–30 kg/m^3^ in gilthead sea bream (*Sparus aurata*) and European sea bass (*Dicentrarchus labrax*) (Person-Le Ruyet and Le Bayon, 2009; Carbonara et al., 2019). However, the occurrence of different stress coping styles plays a key role on how rearing density influences welfare-related responses, being generally accepted that fish that respond in a shy and subordinate manner (reactive fish) are more able to cope with higher densities, while individuals that are bold and aggressive (proactive fish) are more able to cope with low densities (Carbonara et al., 2019). In addition, reduced oxygen (O_2_) availability exacerbates the negative impact of high stocking density on feed intake and growth in gilthead sea bream, triggering different adaptive responses mediated by changes in the gene expression profile of tissue-specific markers of antioxidant defense, oxidative phosphorylation, protein accretion (muscle growth), and lipid metabolism (Martos-Sitcha et al., 2019a).

In gilthead sea bream, there is also now evidence that groups of proactive and reactive individuals did not exhibit consistent escape behavior responses when fish were subjected to restraining tests, which might be indicative that the social context in which fish are kept has an impact on the manifestation of certain personality traits of individuals (Castanheira et al., 2016). Indeed, animal welfare applies to its positive physical and mental condition (Ashley, 2007), and there is now evidence that both swimming activity and schooling behavior are reinforced by high stocking densities in gilthead sea bream (Carbonara et al., 2019; Arechavala-Lopez et al., 2020). Of note, both behavior and swimming performance are also regulated genetically in this species (Perera et al., 2021; Calduch-Giner et al., 2023). At the same time, swimming performance can be improved by a mild-hypoxia pre-conditioning, which leads to a persistent higher critical speed at exercise exhaustion that shifts towards a higher anaerobic fitness following normoxia restoration (Naya-Català et al., 2021). It must be also taken into account that aquaculture finishing production cycle is often accounted during the summer period with relatively high stocking densities and reduced O_2_ availability, and there is still a lack of practical procedures for assessing changes in behavior, physiological traits and welfare indicators in order to fulfill growth performance and welfare status with intensive aquaculture production. Thus, the present study aims to contribute to solve this gap of knowledge, combining the use of AEFishBIT data-loggers for accurate monitoring of swimming activity and breathing rates (Calduch-Giner et al., 2022) with customized PCR-arrays for the simultaneous gene expression profiling of stress-responsive genes (Calduch-Giner et al., 2010; Magnoni et al., 2017; Martos-Sitcha et al., 2019a; Naya-Català et al., 2021), and welfare scores of muscle fat content, blood biochemistry, and external tissue damage (Sánchez-Muros et al., 2013; Seibel et al., 2021; Weirup et al., 2022). The hypothesis of work is that such integrative approach can contribute to better determine the golden stocking density in a given farming condition, encompassing animal welfare and industry interests, with the double aim of mitigating drawback effects but also recognizing the importance of indicators of positive welfare of both physical health and appropriate social interactions (Ashley, 2007; Toni et al., 2019).

## 2 Materials and methods

### 2.1 Ethics

All procedures were approved by the Ethics and Animal Welfare Committee of the Institute of Aquaculture Torre de la Sal (IATS), CSIC Ethics Comittee (permission 1295/2022) and Generalitat Valenciana (permission 2022-VSC-PEA-0230). They were carried out in the IATS’s registered aquaculture infrastructure facility (code ES120330001055) in accordance with the principles published in the European Animal Directive (2010/63/EU) and Spanish laws (Royal Decree RD53/2013) for the protection of animals used in scientific experiments.

### 2.2 Experimental setup

**FIGURE 1:**
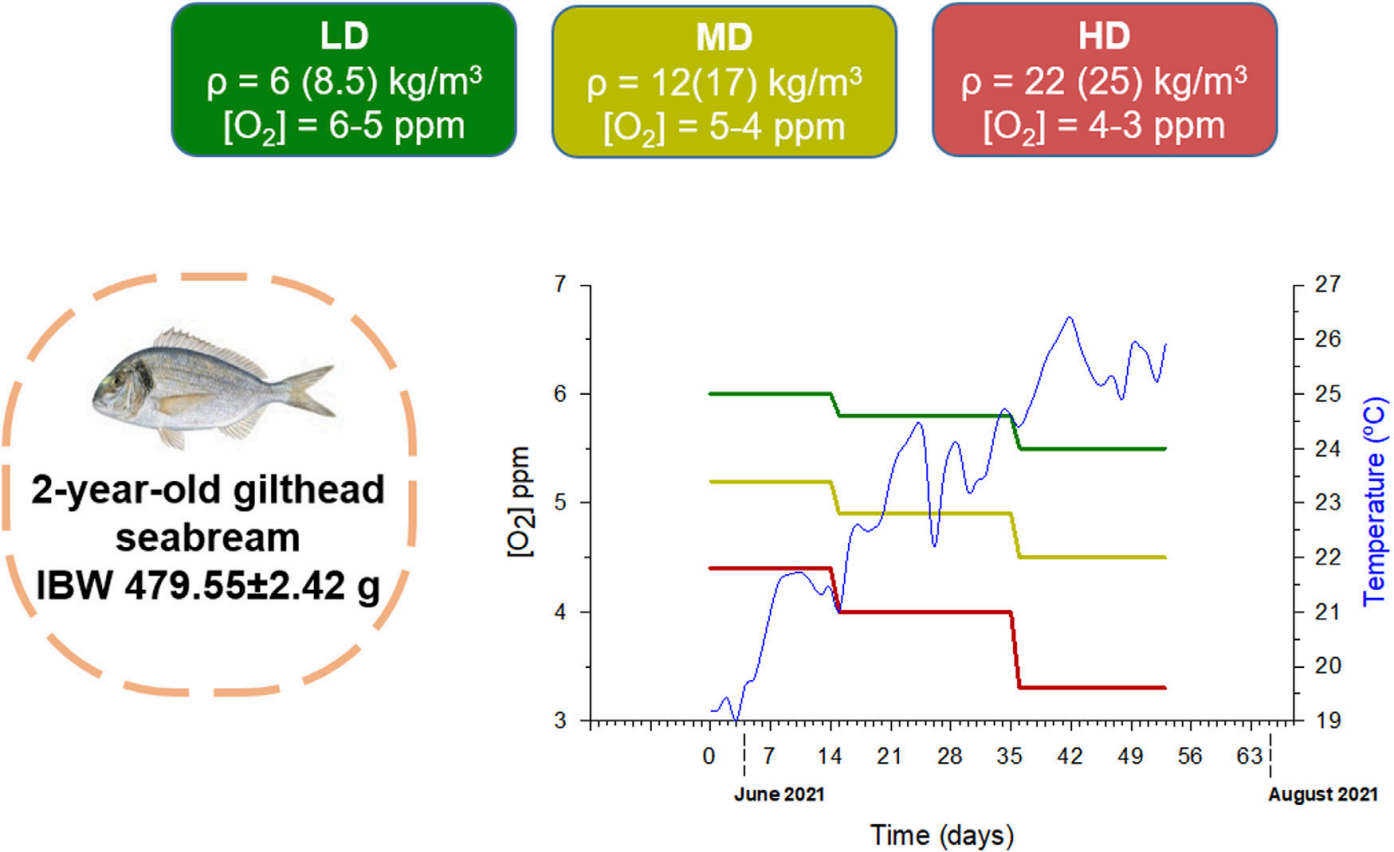
Experimental design of gilthead sea bream reared at three different stocking densities (LD—low density, MD—medium density, HD—high density) and dissolved oxygen concentrations during the summer period. Initial stocking density, ρ, and final density (in parenthesis) is shown. Temporal variation (over 53 days experimental period) in dissolved oxygen concentration for each group and temperature are represented. IBW—Initial body weight; FBW—Final body weight.

Two year-old gilthead sea bream of Mediterranean origin (Avramar, Burriana, Spain) were grown in 3,000L tanks from January to May (12–16 kg/m^3^) in a flow-through system under the natural photoperiod and temperature conditions at the IATS latitude (40° 5′N; 0° 10′E). At the end of May (Figure 1), 462 fish (initial body weight 479.55 ± 2.42 g) were anesthetized with 0.1 g/L MS-222 (Sigma, Saint Louis, MO, United States) and pit-tagged in the dorsal musculature with passive integrated transponders (ID-100A 1.25 Nano Transponder; Trovan, Madrid, Spain). Fish were then re-allocated in experimental tanks (two replicates per condition) at three different stocking densities (low density-LD: 36 fish per tank, 6 kg/m^3^; medium density-MD: 72 fish per tank, 12 kg/m^3^; high density-HD: 123 fish per tank, 22 kg/m^3^), until reaching final stocking densities of 8.5 kg/m^3^ (LD), 17 kg/m^3^ (MD) and 25 kg/m^3^ (HD) after 53 days (mid-July). Body weight and furcal length were recorded at the beginning and at the end of trial, using a FR-200 FishReader W (Trovan, Madrid, Spain). Over the experimental period (May–July, 53 days), fish were fed once daily at a fixed time (12:00 a.m.) with automated feeders near to visual satiety with a standard commercial diet (EFICO 3053, BioMar, Palencia, Spain). Water aeration and flux of inlet water was daily regulated to maintain differentially controlled the water O_2_ concentration (LD, 5–6 ppm, 70%–95% saturation; MD, 4–5 ppm, 55%–75% saturation; HD, 3–4 ppm, 45%–60% saturation). Temperature and water O_2_ concentration was continuously measured through an online environmental monitoring system. Weekly determinations of unionized ammonia were always below the toxic threshold level (<0.05 mg/L).

### 2.3 Behavioral monitoring: physical activity and respiratory frequency

At the end of trial, 12 randomly selected fish per experimental condition (*n* = 6 per replicate tank) were anesthetized with 0.1 g/L MS-222, AEFishBIT devices were externally attached to the operculum for the simultaneous monitoring of physical activity and respiratory frequency, and returned to their original tanks. AEFishBIT is a stand-alone, small, and lightweight motion embedded-microsystem with a tri-axial accelerometer that, by means of attachment to the operculum, monitors physical activity by mapping of the accelerations in *X*- and *Y*-axes, while the operculum beats (*Z*-axis) serve as a measure of respiratory frequency (Calduch-Giner et al., 2022). The devices were programmed for data acquisition of physical activity and respiratory frequency during 2 min every 15 min along two consecutive days, in which fish remained unfed. The sampling frequency of the AEFishBIT device was 100 Hz, and the software pre-processing of raw data was made as described elsewhere (Martos-Sitcha et al., 2019b; Ferrer et al., 2020). At the end of the recording period (48 h post-tagging), all AEFishBIT devices were successfully recovered and pre-processed data were downloaded for tracking the recorded behavioral traits.

### 2.4 Fish sampling for physiological and external tissue damage indicators

After AEFishBIT retrieval, muscle fat content was determined *in situ* with Distell Fish Fat-meter, FM 692 (Distell Ltd., United Kingdom). Fish were then photographed for the evaluation of indicators of external damage (cataracts, exophthalmia, gill status, fin damage and skin lesions) by using a scoring system from 1 to 5 adapted from Hoyle et al. (2007), where 5 indicates maximum damage (Supplementary Figure S1). Such image welfare scoring was completed with 23 additional fish from each experimental condition (35 fish per experimental condition, 105 in total). Additionally, from AEFishBIT recorded fish, blood was taken from caudal vessels with heparinized syringes, and centrifuged at 3,000 g for 20 min at 4°C. The retrieved plasma was aliquoted and stored at −20°C until glucose and cortisol assays. These sampled fish were then killed by cervical section, and portions of liver and dorsal white skeletal muscle (150–200 mg) were excised and collected in RNA later for its storage at −80°C until RNA extraction for gene expression analyses.

### 2.5 Biochemical and molecular analyses

Plasma glucose was determined using the Invitrogen™ Glucose Colorimetric Detection Kit (Invitrogen, EIAGLUC). Plasma cortisol levels were determined with an enzyme Immunoassay Kit (Arbor Assays, K003-H1W) following the manufacturer’s indications. Tissue RNA was extracted using the MagMAX-96 total RNA isolation kit (Life Technologies) after tissue homogenization in TRI reagent following manufacturers’ instructions. RNA quantity and purity was determined by Nanodrop (Thermo Scientific) with absorbance ratios at 260 nm/280 nm of 1.9–2.1. Reverse transcription (RT) of 500 ng of total RNA was performed with random decamers using the High-Capacity cDNA Archive Kit (Applied Biosystems). RT reactions were incubated for 10 min at 25°C and 2 h at 37°C. Negative control reactions were run without reverse transcriptase. Real-time quantitative PCR was carried out with an Eppendorf Mastercycler Ep Realplex, using 96-well PCR array layouts designed for the simultaneous profiling of 44 selected genes of liver and white skeletal muscle (Table 1). The genes comprised in the liver array included markers of the Gh/Igf system (9), lipid metabolism (14), oxidative metabolism and energy sensing (11), and antioxidant defense (10). The analyzed transcripts of muscle included markers of the Gh/Igf system (10), muscle cell growth (8), immune response (5), oxidative metabolism and energy sensing (12), and antioxidant defense (9). Specific primer pair sequences for liver and muscle are listed in Supplementary Tables S1, S2, respectively. Controls of general PCR performance were included on each array, and all the pipetting operations were performed by means of an EpMotion 5070 Liquid Handling Robot (Eppendorf). Briefly, reverse transcription reactions were diluted to convenient concentrations and the equivalent of 660 pg of total input RNA was used in a 25 μL volume for each PCR reaction. PCR-wells contained a 2× SYBR Green Master Mix (Bio-Rad) and specific primers at a final concentration of 0.9 μM were used to obtain amplicons of 50–150 bp in length. The PCR amplification program consisted of an initial denaturation step at 95°C for 3 min, followed by 40 cycles of denaturation for 15 s at 95°C and annealing/extension for 60 s at 60 °C. The efficiency of the PCR reactions was consistently higher than 90% and similar among all genes. The specificity of the reactions was verified by melting curve analysis (ramping rates of 0.5°C/10 s over a temperature range of 55–95°C), and linearity of serial dilutions of RT reactions. Gene expression was calculated using the delta-delta Ct method (Livak and Schmittgen, 2001). β-actin was tested for gene expression stability (GeNorm software, M score = 0.21), and it was used as housekeeping gene in the samples normalization procedure. For multigene expression analysis, all values in the liver were referenced to the expression levels of *gpx1* in LD fish with an arbitrary assigned value of 1. In muscle, gene expression values were referenced to those of *gpx4* in LD fish with an arbitrary assigned value of 1.

**TABLE 1:** PCR-array layout for liver (*) and white skeletal muscle (^†^) gene expression profiling.

Function	Gene	Symbol	GenBank
Gh/Igf SYSTEM	Growth hormone receptor-type 1	ghr1*^†^	AF438176
	Growth hormone receptor-type 2	ghr2*^†^	AY573601
	Insulin-like growth factor 1	igf1*^†^	AY996779
	Insulin-like growth factor 2	igf2*^†^	AY996778
	Insulin-like growth factor binding protein 1a	igfbp1a*	KM522771
	Insulin-like growth factor binding protein 1b	igfbp1b*	MH577189
	Insulin-like growth factor binding protein 2a	igfbp2a*	MH577190
	Insulin-like growth factor binding protein 2b	igfbp2b*	AF377998
	Insulin-like growth factor binding protein 3a	*igfbp3a* ^†^	MH577191
	Insulin-like growth factor binding protein 3b	*igfbp3b* ^†^	MH577192
	Insulin-like growth factor binding protein 4	igfbp4*	KM658998
	Insulin-like growth factor binding protein 5a	*igfbp5a* ^†^	MH577193
	Insulin-like growth factor binding protein 5b	*igfbp5b* ^†^	MH577194
	Insulin-like growth factor binding protein 6a	*igfbp6a* ^†^	MH577195
	Insulin-like growth factor binding protein 6b	*igfbp6b* ^†^	MH577196
LIPID METABOLISM	Elongation of very long chain fatty acids 1	elovl1*	JX975700
	Elongation of very long chain fatty acids 4	elovl4*	JX975701
	Elongation of very long chain fatty acids 5	elovl5*	AY660879
	Elongation of very long chain fatty acids 6	elovl6*	JX975702
	Fatty acid desaturase 2	fads2*	AY055749
	Stearoyl-CoA desaturase 1a	scd1a*	JQ277703
	Stearoyl-CoA desaturase 1b	scd1b*	JQ277704
	Hepatic lipase	hl*	EU254479
	Lipoprotein lipase	lpl*	AY495672
	Adipose triglyceride lipase	atgl*	JX975711
	85 kDa calcium-independent phospholipase A2	pla2g6*	JX975708
	Cholesterol 7-alpha-monooxygenase	cyp7a1*	KX122017
	Peroxisome proliferator-activated receptor α	pparα*	AY590299
	Peroxisome proliferator-activated receptor γ	pparγ*	AY590304
MUSCLE CELL GROWTH	Myoblast determination protein 1	*myod1* ^†^	AF478568
	Myogenic determination protein 2	*myod2* ^†^	AF478569
	Myogenic factor 5	*myf5* ^†^	JN034420
	Myogenic factor 6	*myf6/herculin* ^†^	JN034421
	Myostatin/Growth differentiation factor 8	*mstn/gdf8* ^†^	AF258448
	Myocyte-specific enhancer factor 2a	*mef2a* ^†^	KM522777
	Myocyte-specific enhancer factor 2c	*mef2c* ^†^	KM522778
	Follistatin	*fst* ^†^	AY544167
IMMUNE RESPONSE	Interleukin 1*β*	*il1β* ^†^	AJ419178
	Interleukin 6	*il6* ^†^	EU244588
	Interleukin 8	*il8* ^†^	JX976619
	Interleukin 10	*il10* ^†^	JX976621
	Interleukin 12 subunit *β*	*il12β* ^†^	JX976624
OXIDATIVE METABOLISM & ENERGY SENSING	Hypoxia inducible factor 1*α*	hif1α*^†^	JQ308830
	Proliferator-activated receptor γ coactivator 1*α*	pgc1α*^†^	JX975264
	Proliferator-activated receptor γ coactivator 1*β*	*pgc1β* ^†^	JX975265
	Carnitine palmitoyltransferase 1a	cpt1a*^†^	JQ308822
	Fatty acid binding protein, heart	hfabp*	JQ308834
	Citrate synthase	cs*^†^	JX975229
	NADH-ubiquinone oxidoreductase chain 2	nd2*^†^	KC217558
	NADH-ubiquinone oxidoreductase chain 5	nd5*^†^	KC217559
	Cytochrome c oxidase subunit 1	cox1*^†^	KC217652
	Cytochrome c oxidase subunit 2	cox2*^†^	KC217653
	Uncoupling protein 1	ucp1*	FJ710211
	Uncoupling protein 3	*ucp3* ^†^	EU555336
	Sirtuin1	sirt1*^†^	KF018666
	Sirtuin2	sirt2*^†^	KF018667
ANTIOXIDANT DEFENSE	Catalase	*cat* ^†^	JQ308823
	Glutathione peroxidase 1	gpx1*	DQ524992
	Glutathione peroxidase 4	gpx4*^†^	AM977818
	Glutathione reductase	*gr* ^†^	AJ937873
	Peroxiredoxin 3	prdx3*^†^	GQ252681
	Peroxiredoxin 5	prdx5*^†^	GQ252683
	Superoxide dismutase [Cu-Zn]	cu-zn-sod/sod1*	JQ308832
	Superoxide dismutase [Mn]	mn-sod/sod2*^†^	JQ308833
	Glucose-regulated protein 170 kDa	grp170*^†^	JQ308821
	Glucose-regulated protein 94 kDa	grp94*^†^	JQ308820
	Glucose-regulated protein 75 kDa	grp75*^†^	DQ524993

### 2.6 Statistical analysis

AEFishBIT data were post-processed using a simple cosinor model, which fitted the achieved measurements to a one-harmonic sinusoidal function (Refinetti et al., 2007). Recorded data from incomplete light and dark phases were excluded to avoid any temporal bias. Statistically significant differences (*p* < 0.05) on cosinor-derived data, external damage, blood biochemistry and tissue gene expression were assessed by one-way ANOVA followed by a Holm-Sidak *post hoc* test, using the SigmaPlot software 14.5 (Systat Software, San Jose, CA, United States). Correlation analysis was also assessed using the SigmaPlot software. Graphical representations of gathered biomarkers networks was made with the Cytoscape v3.9.1 software. Gene expression patterns were further analyzed by partial least-squares discriminant analysis (PLS-DA) using EZinfo v3.0 (Umetrics, Umeå, Sweden). The quality of the PLS-DA model was evaluated by the parameters R2Y (cum) and Q2 (cum), which indicate the fit and prediction ability, respectively. A validation test of the PLS-DA model consisting of 500 random permutations (Ojala and Garriga, 2010) was performed using the Bioconductor R package *ropls* (Thévenot et al., 2015). The list of genes contributing to group separation was determined by the minimum Variable Importance in the Projection (VIP) values. Discriminant genes were considered with a VIP threshold ≥1.0 (Li et al., 2012; Kieffer et al., 2016).

## 3 Results

### 3.1 Welfare scores of growth performance, external damage and blood stress markers

As shown in Table 2, the initial body weight (474–484 g) did not differ significantly among the three experimental groups. At the end of trial, the mean body weight of HD fish (598 g) was significantly lower than in MD and LD fish (662 and 669 g, respectively). In parallel, feed intake was significantly higher in LD and MD fish than in HD fish, and specific growth rates (SGR) in HD fish highlighted a 40% reduction (SGR HD, 0.39%; SGR LD-MD, 0.62%–0.66%). Fulton´s body condition factor K (CFK) ranged from 2.95 in LD fish to 2.72 in HD fish. These observations paralleled with a significant increase of feed conversion ratio (FCR) from 1.61–1.66 in LD-MD fish to 1.92 in HD fish. A concurrent decrease of liver weight and hepatosomatic index (HSI) was also found with the increase of stocking density, varying the achieved HSI from 1.17 to 1.14 in LD-MD fish to 0.91 in HD fish. Additionally, a slight (not statistically significant) decrease in muscle fat content was observed in HD fish, pointing out a leaner body shape with a decreased CFK. Regarding blood biochemical parameters, plasma glucose levels were significantly higher in HD fish (108 mg/dL) than in LD fish (89.5 mg/dL), with intermediate values in the MD group that was closer to HD rather than LD fish. Plasma cortisol levels showed the same trend, and the measured values in HD and MD fish (114–106 ng/mL) were significantly higher than in LD fish (64 ng/mL).

**TABLE 2:** Data on growth performance and basic blood biochemistry of gilthead sea bream reared at three different stocking densities (final density, 8.5–25 kg/m^3^) from end of May to mid-July 2021 (53 days). Data on whole body biometrics, feed intake and feed conversion are the mean ± SEM of duplicated tanks. Liver weight, hepatosomatic index, muscle fat content, and plasma cortisol and metabolite levels are the mean ± SEM of 12 fish per experimental condition. Different letters indicate statistically significant differences (Holm-Sidak *post hoc* test, *p* < 0.05).

	LD (8.5)	MD (17)	HD (25)	*P* ^a^
Initial body weight (g)	474.7 ± 8.87	474.3 ± 6.55	484.1 ± 4.48	0.379
Initial body length (cm)	25.58 ± 0.16	25.74 ± 0.11	25.86 ± 0.08	0.243
Feed intake (g DM/fish)	313.80 ± 2.55^a^	310.51 ± 2.31^a^	220.28 ± 2.40^b^	<0.001
Final body weight (g)	669.33 ± 11.6^a^	661.55 ± 9.41^a^	598.39 ± 6.51^b^	<0.001
Final body length (cm)	28.33 ± 0.18^ab^	28.69 ± 0.13^a^	28.19 ± 0.09^b^	0.008
Final CFK^b^	2.95 ± 0.05^a^	2.80 ± 0.03^ab^	2.72 ± 0.06^b^	0.041
Liver weight (g)	8.17 ± 0.66^a^	7.75 ± 0.52^a^	5.89 ± 0.38^b^	0.011
HSI (%)^c^	1.17 ± 0.09^a^	1.14 ± 0.04^a^	0.91 ± 0.05^b^	0.017
SGR (%)^d^	0.66 ± 0.03^a^	0.62 ± 0.01^a^	0.39 ± 0.01^b^	<0.001
FCR^e^	1.61 ± 0.06^b^	1.66 ± 0.04^b^	1.92 ± 0.04^a^	0.036
Glucose (mg/dL)	89.48 ± 6.02^b^	101.48 ± 7.23^ab^	107.85 ± 5.60^a^	0.036
Cortisol (ng/mL)	64.29 ± 8.69^b^	105.79 ± 10.59^a^	114.25 ± 11.50^a^	0.004
Muscle fat (%)^f^	9.88 ± 0.28	10.16 ± 0.38	9.28 ± 0.45	0.257

^a^ One-way ANOVA *p-value*.

^b^ Fulton’s body condition factor, CFK, 100 x (body weight/standard length^c^).

^c^ Hepatosomatic index, HSI = 100 × (liver weight/fish weight).

^d^ Specific growth rate, SGR = 100 × (ln final body weight—ln initial body weight)/days.

^e^ Feed conversion ratio, FCR = 100 × (dry feed intake/wet weight gain).

^f^ Fatmeter measurements.

**FIGURE 2:**
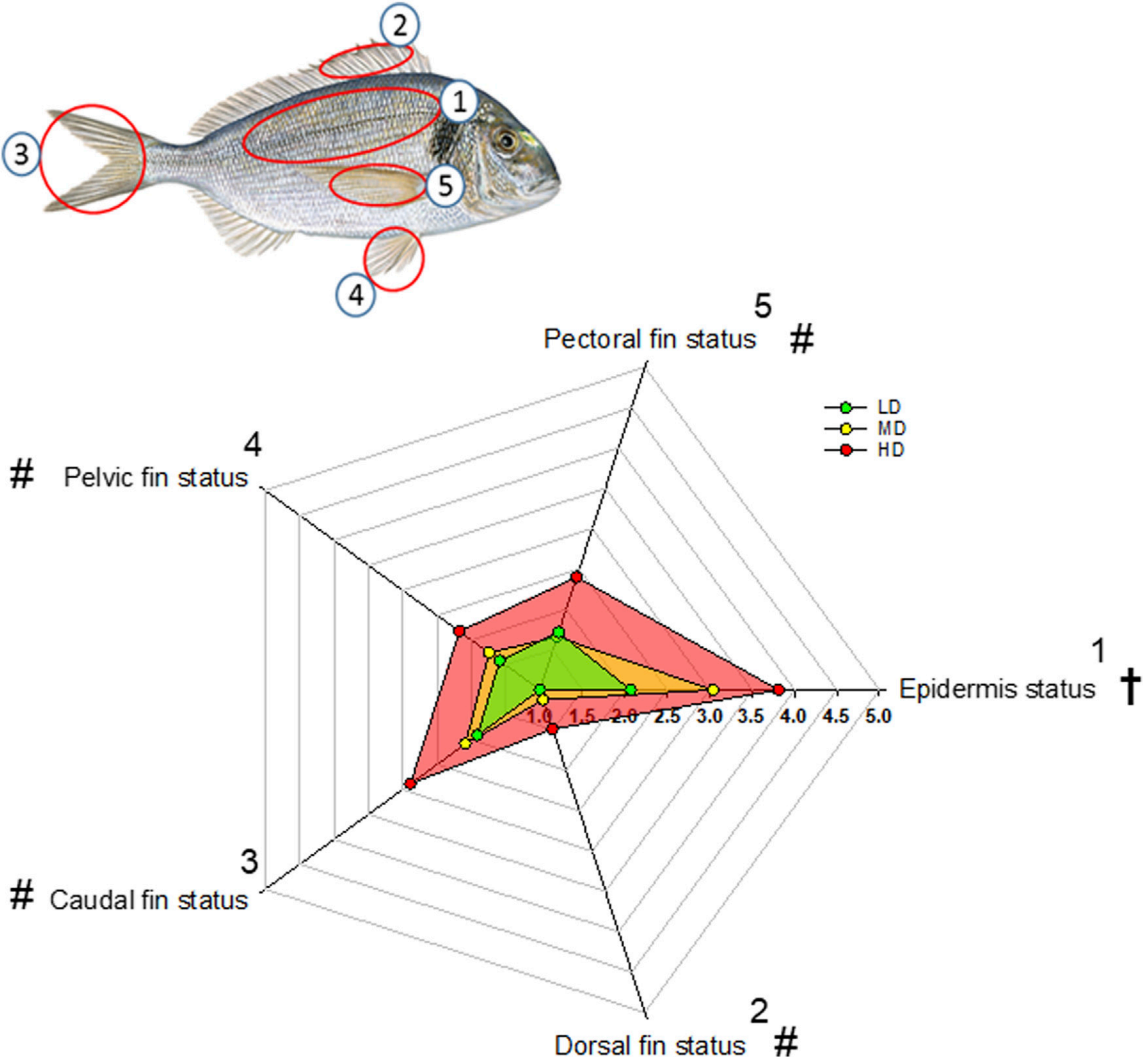
Radar plot representing external welfare indicators of gilthead sea bream reared at three different densities (scoring system from 1 to 5 adapted from Hoyle et al. (2007). Numbered fish body parts are indicated for visualization. Colored points are the mean (*n* = 35) of each welfare indicator. Symbols, † and #, indicate significant differences between the three density groups and between HD and the other two experimental densities, respectively, (*p* < 0.05, ANOVA, Holm-Sidak test).

Scores of indicators of external damage are represented in the Radar Plot of Figure 2. Epidermal status was progressively impaired with the increase of stocking density and the worst status was clearly achieved in fish held at HD. Dorsal, caudal, pelvic and pectoral fin status were also affected by the stocking density, but in this case HD fish clearly evolved as a differential group in comparison to both LD and MD fish. Signs of cataracts or exophthalmia were absent in all fish groups, and gill status was visually similar and in good condition in all fish.

### 3.2 Behavioral synchronization by high stocking density

**FIGURE 3:**
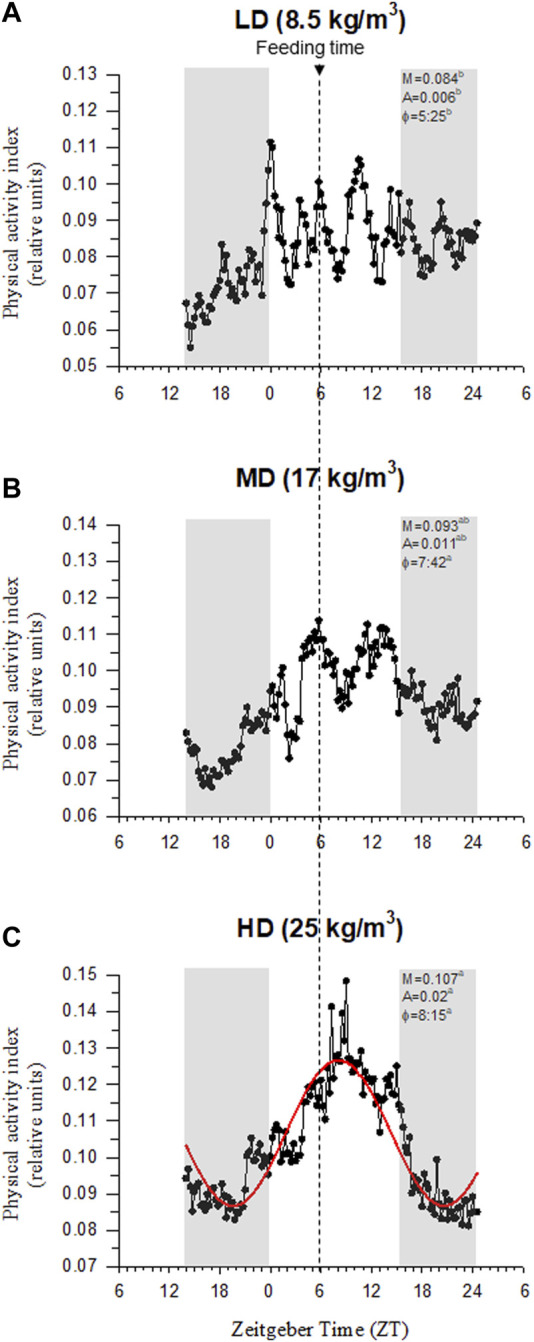
Gilthead sea bream physical activity synchronization by feeding at low density **(A)**, medium density **(B)** and high density **(C)**. AEFishBIT data (measures taken every 15 min along 2 consecutive days) of representative individuals (*n* = 8) is shown as a continuous dotted line in each panel. Best-fit curve (red sinusoidal line) derived from the cosinor analysis of physical activity is only represented in the HD group. Values of mesor (M), amplitude (A), acrophase (φ) and *p*-value (*P*) of best-fit curves are shown for each density. Gray shaded areas represent dark phases. Arrow in vertical dotted line indicates the feeding time. Different letters represent significant (*p* < 0.05, ANOVA, Holm-Sidak test) differences between density groups.

**FIGURE 4:**
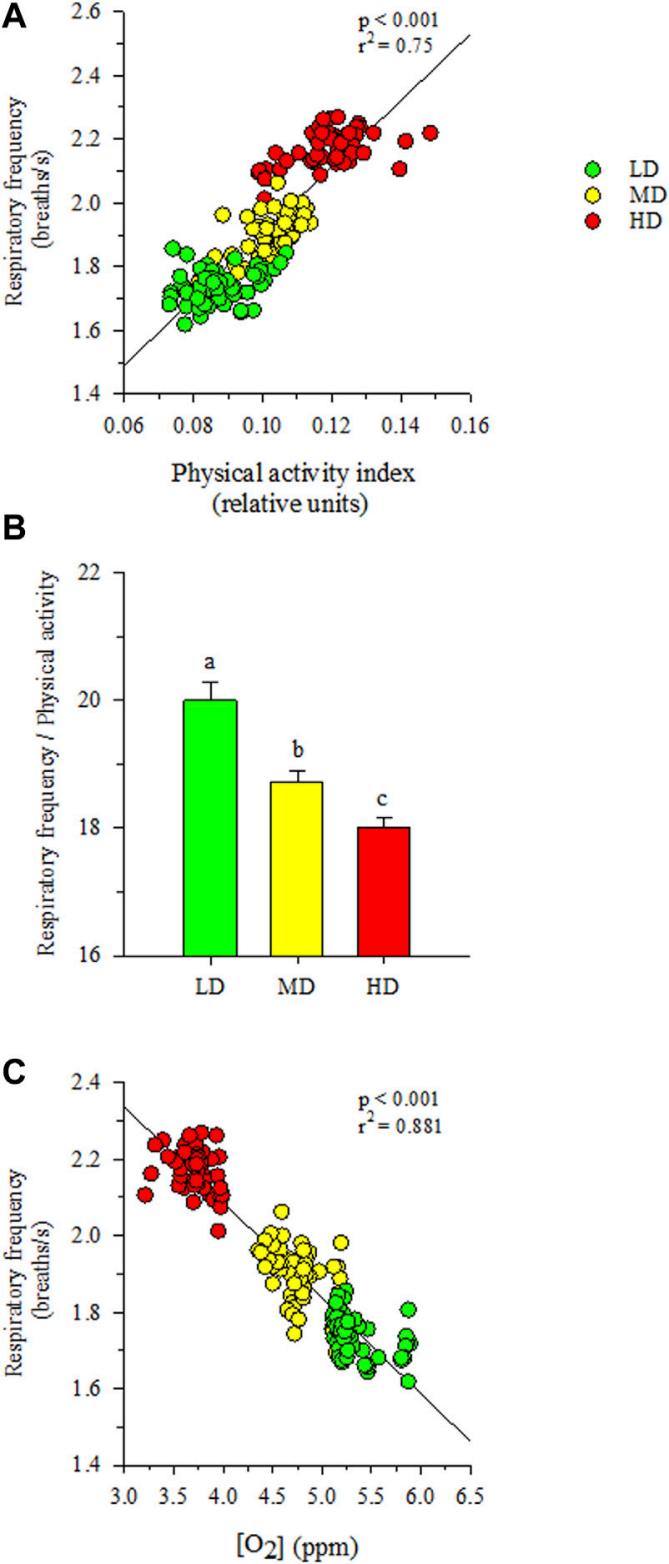
**(A)** Correlation plot of respiratory frequency and physical activity. **(B)** Respiratory frequency/physical activity ratio in each stocking density group. Different letters represent significant (*p* < 0.05, ANOVA, Holm-Sidak test) differences between groups. **(C)** Correlation plot of respiratory frequency and water dissolved oxygen concentration. Values are the mean of physical activity and/or respiratory frequency (*n* = 8) of each density treatment and dissolved oxygen concentration each 15 min during the diurnal period.

Visual observations highlighted a more heterogeneous swimming behavior in fish held at low densities than in HD fish. Certainly, the recorded physical activity and respiratory frequency fitted better to the cosinor model in HD fish than in the other two experimental groups, which became especially evident in the case of LD fish (Supplementary Figure S2). This would reflect an improved social cohesion in HD fish, acting the feeding time as a major zeitgeber synchronization factor with the acrophase of physical activity (φ, the time period in which the cycle peaks) clearly surrounding the programmed feeding time in the absence of feed provision (Figure 3). The rhythm of activity in HD fish also disclosed a higher adjusted mean (M, mesor) and a wide range of variation (A, amplitude), which became statistically significant when the comparison was made with the other extreme group (LD fish) (intermediate values were achieved in MD fish). Over the entire diurnal recording period (09:00 to 21:00 h), correlation analyses highlighted a close positive lineal correlation (*p* < 0.001) between physical activity and respiratory frequency for all tracked fish considered as a whole (Figure 4A), which in turn rendered a decreased respiration/activity ratio with the increase of stocking densities (Figure 4B). With independence of this, the respiration tracking rendered a strong negative correlation (*p* < 0.001) with the continuously recorded water O_2_ concentration (Figure 4C).

### 3.3 Tissue-specific gene expression patterns

**FIGURE 5:**
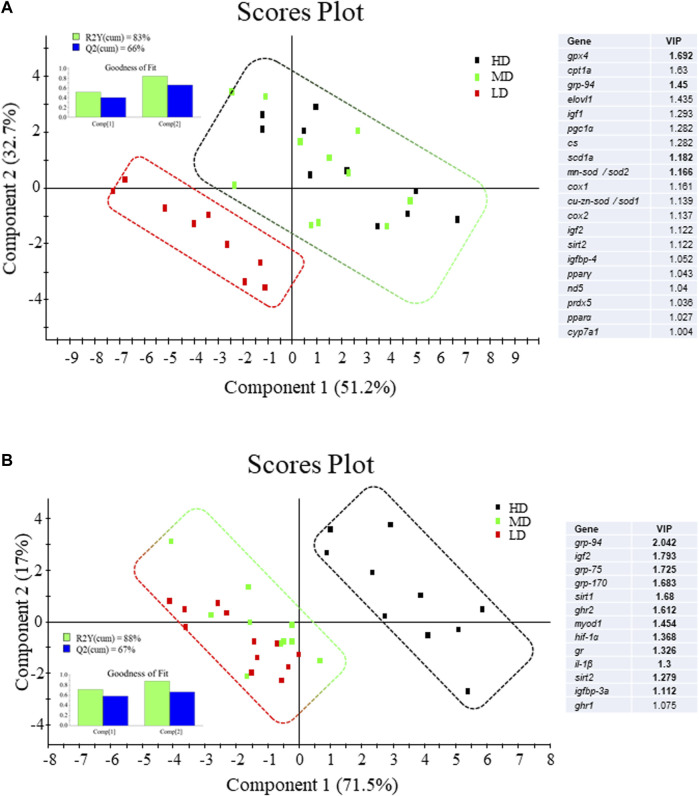
Two-dimensional PLS-DA score plot of **(A)** liver and **(B)** white skeletal muscle molecular signatures of fish under different stocking densities, representing the distribution of the samples between the first two components in the model. Top left inserted figures represent the cumulative explained [R2Y(cum), green bars] and predicted [Q2(cum), blue bars] variance. Blue tables represent the ordered list of markers by variable importance in the projection (VIP) of the PLS-DA model. Upregulated genes are indicated by VIP values in bold and downregulated genes are shown as VIP values in regular font.

All the genes in the liver and muscle PCR-arrays were expressed at detectable levels. In liver, up to 10 genes were differentially expressed by One-way ANOVA (Table 3), being 6 downregulated (*igf1*, *igf2, cyp7a1*, *pparα, cox1*, *cox2*) and 4 upregulated (*igfbp1a, scd1a, mn-sod/sod2*, *grp94)* in HD fish in comparison to LD fish. The hepatic expression pattern of MD fish was related to HD fish rather than LD fish, and PLS-DA was able to differentiate LD fish and MD-HD fish with 20 genes out of 44 of discriminant value (VIP >1.0) (Figure 5A). Among them, up to 16 were downregulated in HD-MD fish. The fitness and predictability of the PLS-DA model was validated by a 500-random permutation test (Supplementary Figure S3A) (*p* < 0.05), explaining the two first components the 83% and 66% of the observed and predicted variance, respectively.

**TABLE 3:** Relative gene expression of liver mRNA transcripts of fish reared at three different stocking densities (final density; 8.5-17-25 kg/m^3^). Values are the mean ± SEM of 10–12 fish per experimental condition. All data are in reference to the expression level of *gpx1* in fish from LD group with an arbitrary value of 1. Different letters indicate statistically significant differences (Holm-Sidak *post hoc* test, *p* < 0.05). Differentially expressed genes are in bold.

	LD (8.5)	MD (17)	HD (25)	*P* ^a^
*ghr1*	1.84 ± 0.13	1.78 ± 0.12	1.84 ± 0.14	0.927
*ghr2*	1.16 ± 0.15	1.14 ± 0.09	1.08 ± 0.11	0.88
***igf1***	9.92 ± 0.97^a^	7.22 ± 0.58^b^	6.73 ± 0.55^b^	0.008
***igf2***	3.53 ± 0.63^a^	1.85 ± 0.17^b^	1.86 ± 0.27^b^	0.008
***igfbp1a***	0.05 ± 0.01^b^	0.12 ± 0.03^ab^	0.22 ± 0.07^a^	0.039
*igfbp1b*	2.44 ± 0.52	2.43 ± 0.78	2.23 ± 0.69	0.969
*igfbp2a*	0.62 ± 0.07	0.51 ± 0.05	0.46 ± 0.05	0.219
*igfbp2b*	1.27 ± 0.14	1.30 ± 0.14	1.27 ± 0.10	0.976
*igfbp4*	0.63 ± 0.06	0.57 ± 0.05	0.50 ± 0.04	0.219
*elovl1*	5.37 ± 0.48	4.43 ± 0.83	3.82 ± 0.42	0.203
*elovl4*	0.18 ± 0.01	0.22 ± 0.03	0.21 ± 0.02	0.465
*elovl5*	0.88 ± 0.18	1.25 ± 0.23	1.14 ± 0.20	0.448
*elovl6*	1.22 ± 0.15	1.26 ± 0.15	1.07 ± 0.23	0.738
*fads2*	0.36 ± 0.06	0.42 ± 0.12	0.32 ± 0.05	0.71
***scd1a***	0.09 ± 0.01^b^	0.11 ± 0.01^ab^	0.16 ± 0.03^a^	0.026
*scd1b*	0.26 ± 0.05	0.41 ± 0.08	0.37 ± 0.17	0.594
*hl*	4.77 ± 0.41	4.72 ± 0.43	4.58 ± 0.24	0.929
*lpl*	2.19 ± 0.29	1.94 ± 0.32	2.44 ± 0.30	0.519
*atgl*	0.29 ± 0.11	0.34 ± 0.10	0.24 ± 0.06	0.753
*pla2g6*	0.09 ± 0.01	0.10 ± 0.01	0.10 ± 0.01	0.43
***cyp7a1***	1.40 ± 0.22^a^	0.96 ± 0.16^ab^	0.70 ± 0.11^b^	0.02
***pparα***	1.83 ± 0.16^a^	1.66 ± 0.11^ab^	1.37 ± 0.09^b^	0.048
*pparγ*	0.28 ± 0.01	0.24 ± 0.01	0.25 ± 0.03	0.321
*hif1α*	0.40 ± 0.02	0.38 ± 0.03	0.41 ± 0.02	0.722
*pgc1α*	0.06 ± 0.01	0.03 ± 0.01	0.05 ± 0.01	0.397
*cpt1a*	0.45 ± 0.04	0.37 ± 0.07	0.30 ± 0.04	0.133
*hfabp*	20.83 ± 1.72	20.62 ± 2.62	18.92 ± 1.82	0.779
*Cs*	0.49 ± 0.03	0.47 ± 0.10	0.38 ± 0.03	0.406
*nd2*	15.16 ± 2.27	12.90 ± 2.90	10.46 ± 1.51	0.34
*nd5*	5.56 ± 0.65	4.32 ± 0.38	4.31 ± 0.47	0.157
***cox1***	58.13 ± 8.60^a^	45.31 ± 3.92^ab^	36.02 ± 3.84^b^	0.041
***cox2***	17.74 ± 2.08^a^	13.92 ± 1.38^ab^	10.66 ± 1.20^b^	0.013
*ucp1*	8.84 ± 1.00	7.05 ± 0.77	8.42 ± 1.13	0.407
*sirt1*	0.06 ± 0.00	0.05 ± 0.01	0.06 ± 0.00	0.896
*sirt2*	0.16 ± 0.01	0.16 ± 0.01	0.15 ± 0.01	0.628
*gpx1*	1.04 ± 0.08	0.93 ± 0.08	1.11 ± 0.09	0.356
*gpx4*	4.56 ± 0.31	6.09 ± 0.49	5.71 ± 0.52	0.057
*prdx3*	0.52 ± 0.04	0.42 ± 0.05	0.49 ± 0.04	0.279
*prdx5*	0.62 ± 0.05	0.54 ± 0.04	0.52 ± 0.05	0.31
*cu-zn-sod/sod1*	3.23 ± 0.22	3.13 ± 0.27	2.87 ± 0.22	0.55
***mn-sod/sod2***	0.56 ± 0.03^b^	0.60 ± 0.03^ab^	0.75 ± 0.07^a^	0.031
*grp170*	0.55 ± 0.06	0.74 ± 0.19	0.80 ± 0.18	0.486
***grp94***	1.74 ± 0.16^b^	3.00 ± 0.31^ab^	4.09 ± 0.82^a^	0.01
*grp75*	0.39 ± 0.03	0.28 ± 0.02	0.37 ± 0.05	0.079

^a^ One-way ANOVA *p-value*.

Gene expression profiling of white skeletal muscle displayed significant differences among experimental groups with 9 upregulated genes (*ghr2*, *igf2*, *igfbp3a, il1β*, *sirt1*, *sirt2, grp170*, *grp94*, *grp75*) and one downregulated gene (*ghr1*) in HD fish by One-way ANOVA (Table 4). However, in this case, MD fish was related to LD fish rather than HD fish, and PLS-DA was able to differentiate HD fish from MD-LD fish with 13 genes out of 44 of discriminant value (VIP >1.0). Among them, up to 12 were upregulated in HD fish (Figure 5B). The fitness and predictability of the PLS-DA model was validated by a 500-random permutation test (Supplementary Figure S3B) (*p* < 0.01), explaining the two first components the 88% and 67% of the observed and predicted variance, respectively.

**TABLE 4:** Relative gene expression of white skeletal muscle mRNA transcripts of fish reared at three different stocking densities (final density; 8.5-17-25 kg/m3). Values are the mean ± SEM of 10–12 fish per experimental condition. All data are in reference to the expression level of *gpx4* in fish from LD group with an arbitrary value of 1. Different letters indicate statistically significant differences (Holm-Sidak *post hoc* test, *p* < 0.05). Differentially expressed genes are in bold.

	LD (8.5)	MD (17)	HD (25)	*P* ^a^
***ghr1***	3.07 ± 0.24^a^	2.54 ± 0.30^ab^	2.12 ± 0.15^b^	0.027
***ghr2***	0.48 ± 0.06^b^	0.44 ± 0.07^b^	0.73 ± 0.08^a^	0.012
*igf1*	0.06 ± 0.01	0.06 ± 0.01	0.08 ± 0.01	0.223
***igf2***	0.53 ± 0.03^b^	0.62 ± 0.06^b^	0.80 ± 0.06^a^	0.003
***igfbp3a***	1.10 ± 0.13^b^	1.26 ± 0.11^ab^	1.53 ± 0.15^a^	0.042
*igfbp3b*	0.004 ± 0.001	0.004 ± 0.001	0.006 ± 0.002	0.257
*igfbp5a*	0.26 ± 0.04	0.29 ± 0.05	0.38 ± 0.06	0.241
*igfbp5b*	2.00 ± 0.22	2.32 ± 0.36	2.56 ± 0.22	0.362
*igfbp6a*	0.020 ± 0.004	0.018 ± 0.003	0.021 ± 0.002	0.773
*igfbp6b*	0.09 ± 0.01	0.10 ± 0.02	0.11 ± 0.02	0.849
*myod1*	4.66 ± 0.33	5.42 ± 0.59	6.10 ± 0.54	0.141
*myod2*	0.96 ± 0.08	1.13 ± 0.08	0.97 ± 0.09	0.301
*myf5*	0.15 ± 0.01	0.18 ± 0.01	0.16 ± 0.01	0.222
*myf6/herculin*	0.19 ± 0.01	0.21 ± 0.02	0.21 ± 0.02	0.52
*mstn/gdf8*	0.80 ± 0.15	1.03 ± 0.18	1.40 ± 0.42	0.336
*mef2a*	7.51 ± 0.54	6.60 ± 0.39	7.14 ± 0.44	0.387
*mef2c*	2.42 ± 0.10	2.21 ± 0.14	2.12 ± 0.16	0.27
*fst*	0.29 ± 0.03	0.31 ± 0.05	0.30 ± 0.03	0.955
***il1β***	0.051 ± 0.005^b^	0.085 ± 0.011^ab^	0.106 ± 0.016^a^	0.005
*il6*	0.004 ± 0.001	0.003 ± 0.000	0.004 ± 0.001	0.311
*il8*	0.012 ± 0.002	0.017 ± 0.002	0.020 ± 0.003	0.115
*il10*	0.006 ± 0.001	0.008 ± 0.001	0.008 ± 0.001	0.472
*il12β*	0.012 ± 0.001	0.011 ± 0.001	0.015 ± 0.001	0.151
*hif1α*	0.70 ± 0.05	0.78 ± 0.07	0.89 ± 0.06	0.086
*pgc1α*	0.89 ± 0.15	0.60 ± 0.12	0.61 ± 0.17	0.313
*pgc1β*	0.44 ± 0.04	0.40 ± 0.04	0.38 ± 0.03	0.542
*cpt1a*	2.22 ± 0.14	2.33 ± 0.19	2.42 ± 0.14	0.656
*cs*	8.39 ± 0.41	8.00 ± 0.51	8.12 ± 0.44	0.83
*nd2*	37.93 ± 2.67	37.89 ± 4.35	32.01 ± 3.03	0.383
*nd5*	12.88 ± 1.03	12.06 ± 0.94	11.16 ± 1.09	0.5
*cox1*	184.30 ± 14.18	171.38 ± 10.78	176.18 ± 8.87	0.726
*cox2*	34.06 ± 3.45	35.82 ± 2.48	34.46 ± 2.57	0.903
*ucp3*	6.68 ± 0.61	5.29 ± 0.56	5.36 ± 0.58	0.175
***sirt1***	0.21 ± 0.01^b^	0.24 ± 0.02^ab^	0.28 ± 0.01^a^	0.009
***sirt2***	0.46 ± 0.02^b^	0.50 ± 0.03^ab^	0.59 ± 0.04^a^	0.016
*cat*	4.81 ± 0.17	5.02 ± 0.34	5.33 ± 0.29	0.413
*gpx4*	1.00 ± 0.27	1.02 ± 0.27	1.50 ± 0.37	0.432
*gr*	0.20 ± 0.01	0.23 ± 0.02	0.25 ± 0.02	0.061
*prdx3*	1.87 ± 0.13	1.94 ± 0.13	2.16 ± 0.14	0.293
*prdx5*	6.33 ± 0.36	6.79 ± 0.54	6.13 ± 0.38	0.552
*mn-sod/sod2*	1.90 ± 0.14	1.96 ± 0.15	2.11 ± 0.17	0.601
***grp170***	0.25 ± 0.02^b^	0.27 ± 0.02^b^	0.38 ± 0.03^a^	<0.001
***grp94***	0.72 ± 0.05^b^	0.87 ± 0.08^b^	1.32 ± 0.11^a^	<0.001
***grp75***	1.17 ± 0.05^b^	1.32 ± 0.08^b^	1.63 ± 0.10^a^	<0.001

^a^ One-way ANOVA *p-value*.

### 3.4 Understanding the physiological significance of gathered biomarkers

**FIGURE 6:**
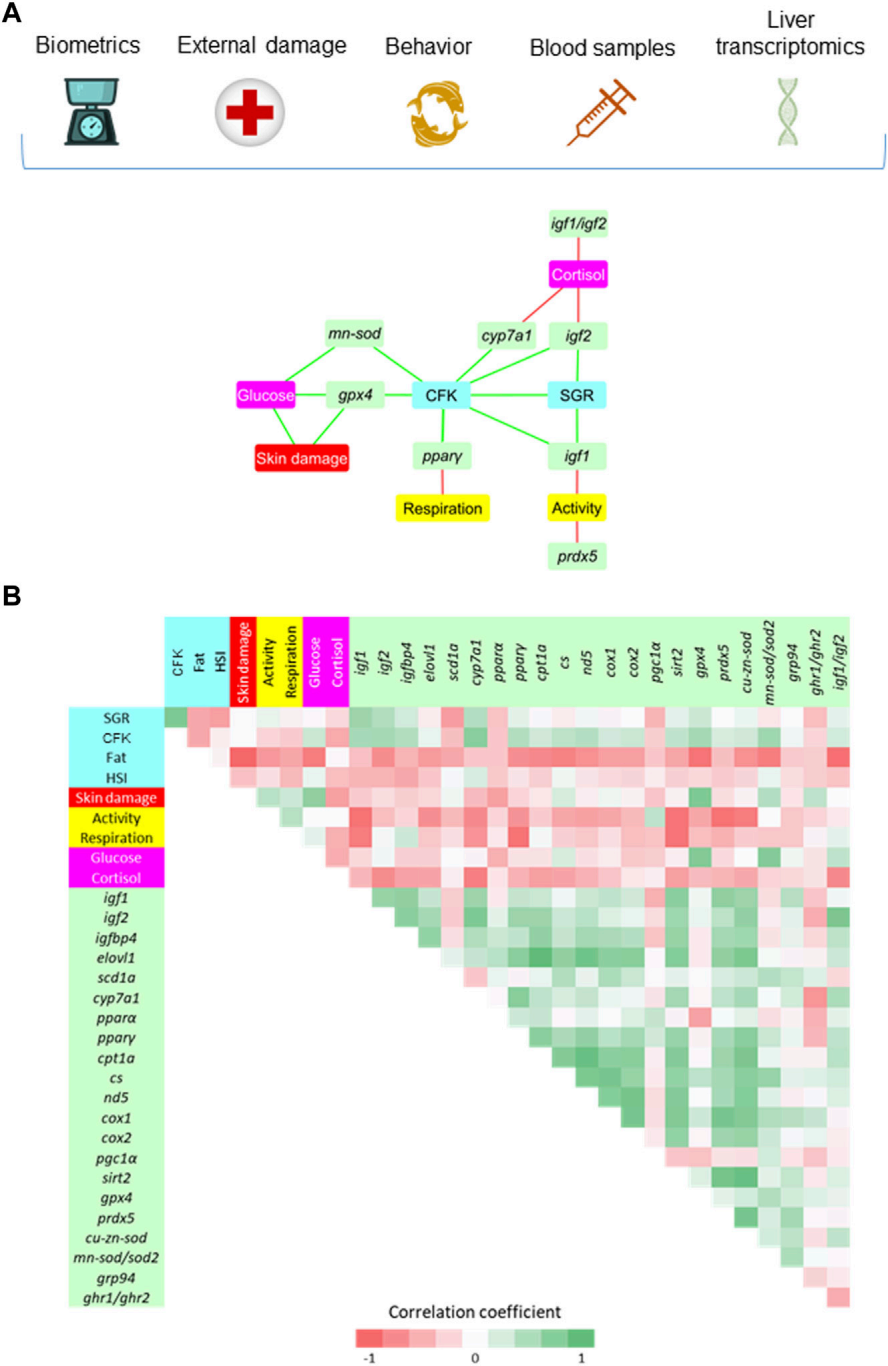
**(A)** Diagram network showing the positive (green) and negative (red) significant (*p* < 0.05) Spearman correlation coefficients (7–12 fish from the HD group) between hepatic genes (VIP > 1), biometric indicators (SGR, CFK, Fat and HSI), external damage (Skin) and behavioral parameters (Activity and Respiration), and blood stress markers (Glucose and Cortisol). **(B)** Correlation matrix showing graphically the entire set of correlation coefficients. The values of Spearman correlation coefficients can be accessed at Supplementary Table S3.

Based on the differential HD outcomes on growth performance, external damage, behavior, plasma stress markers and tissue gene expression, a biomarker network was built to disclose at a glance the tissue-specific adaptive responses of liver and white skeletal muscle in this group of fish. In the case of liver, a total of 19 significant correlations (*p ≤* 0.05) were established linking hepatic discriminant genes with other welfare indicators (Figure 6A). A direct link between CFK and SGR was presented at the center of the hub, being the rest of markers indirectly connected by different discriminant genes. Hence, SGR and CFK were positively correlated with *igf1* and *igf2*, which were at the same time negatively correlated with physical activity and plasma cortisol, respectively. Moreover, plasma cortisol was negatively correlated with *cyp7a1* and the *igf1/igf2* ratio*.* The same trend was found between physical activity and the antioxidant defense gene *prdx5*. The CFK was also positively correlated with other defense antioxidant enzymes (*mn-sod, gpx4*) and lipid metabolism-related genes (*cyp7a1*, *pparγ)*. Lastly, the *pparγ* was negatively correlated with the respiratory frequency, while plasma glucose was positively correlated with signs of skin damage and with *gpx4* and *mn-sod* genes. There was also a positive correlation between skin damage and *gpx4*. The significance of all the gathered interrelationships with hepatic transcripts can be accessed in Supplementary Table S3.

**FIGURE 7:**
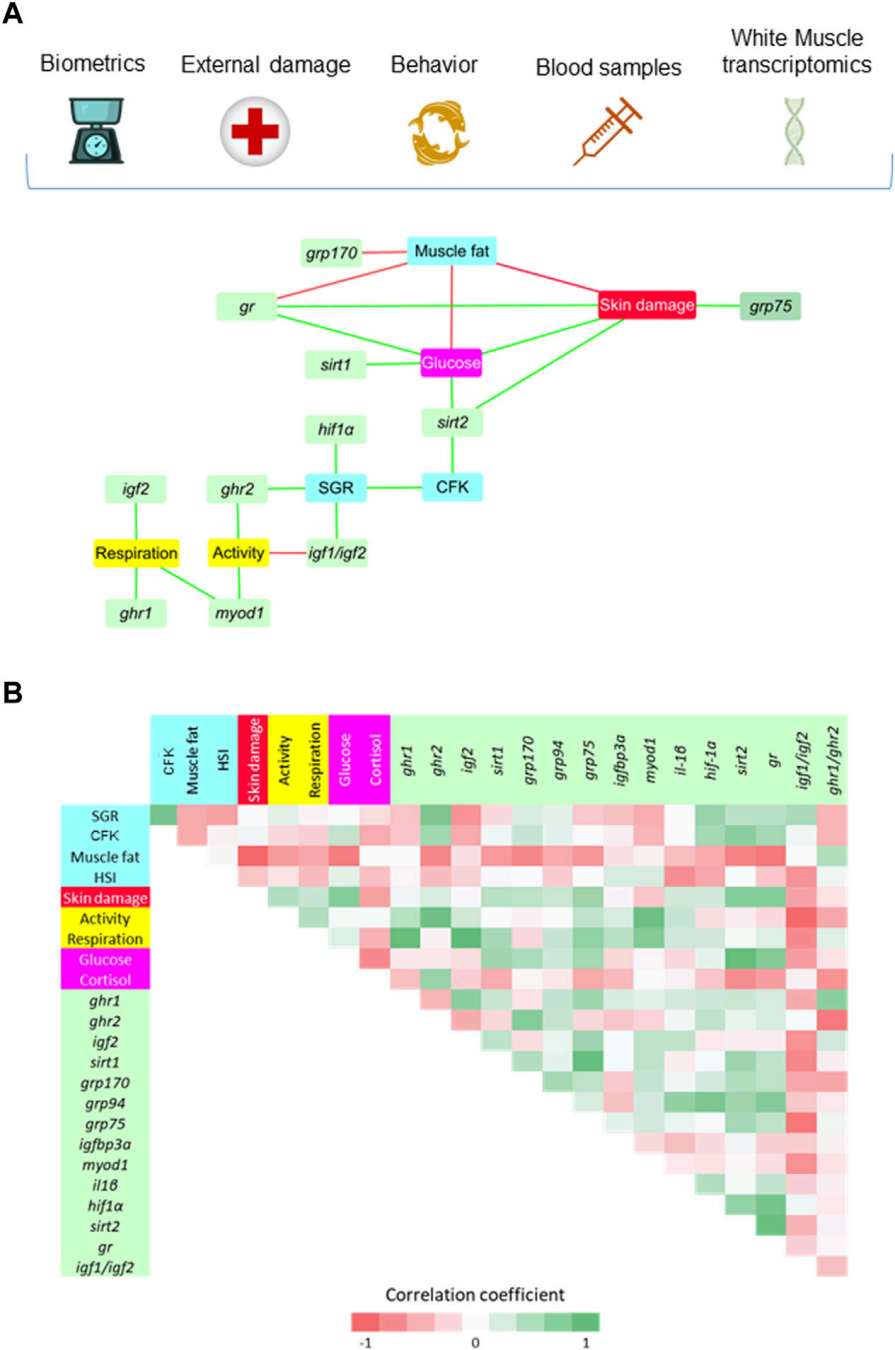
**(A)** Diagram network showing the positive (green) and negative (red) significant (*p* < 0.05) Spearman correlation coefficients (7–12 fish from the HD group) between white skeletal muscle genes (VIP > 1), biometric indicators (SGR, CFK, Fat and HSI), external damage (Skin) and behavioral parameters (Activity and Respiration), and blood stress markers (Glucose and Cortisol). **(B)** Correlation matrix showing graphically the entire set of correlation coefficients. The values of Spearman correlation coefficients can be accessed at Supplementary Table S4.

Regarding the white skeletal muscle, a total of 21 significant correlations (*p <* 0.05) were established among muscle discriminant genes, biometric, behavioral and biochemical blood markers (Figure 7A). As in liver, the SGR-CFK link was indirectly connected to the rest of welfare indicators through discriminant genes. SGR was positively correlated with *hif1α*, *igf1/igf2* ratio and *ghr2*. At the same time, physical activity was indirectly connected to SGR through *ghr2* and *igf1/igf2*, either in a positive or negative manner, respectively. Physical activity was also positively correlated with a muscle cell growth marker (*myod1*), and the same trend was found between *myod1* and respiratory frequency. Genes related to Gh/Igf system (*igf2* and *ghr1*) were also positively correlated with respiration. On the other side of the SGR-CFK link, *sirt2* was positively correlated with CFK, plasma glucose and skin damage. Plasma glucose was negatively correlated with muscle fat content, and positively correlated with *sirt1*, *gr* and skin damage. In a similar way, skin damage was negatively correlated with muscle fat content, and positively correlated with antioxidant defense genes (*gr*, *grp75*). By last, muscle fat content was negatively correlated with *gr* and *grp170*. The significance of all the gathered interrelationships with muscle transcripts can be accessed in Supplementary Table S4.

## 4 Discussion

The negative impact of climate change in fisheries and aquaculture has been extensively studied and reviewed at regional and global scales (FAO, 2020; Maulu et al., 2021). Most studies, however, tend to explore the negative effects of climate change, while giving far less attention to the positive ones that are especially critical for the adaptation strategies. Certainly, warmer periods can promote shorter growing periods, helping to the expansion of aquaculture production in both temperate and cold regions, though such positive achievements become outweighed below the Limiting Oxygen Saturation (LOS) level, defined as the threshold level where regulatory mechanism are no longer sufficient to maintain O_2_ consumption without compromising any physiological function (Remen et al., 2015; Remen et al., 2016). Thus, in the present study, fish stocked at the highest density (25 kg/m^3^, 45%–60% O_2_ saturation level) experienced an impaired growth in comparison to fish reared at low (LD, 8.5 kg/m^3^) or intermediate (MD, 17 kg/m^3^) densities. Similar results were reported by Carbonara et al. (2019) at 15–30 kg/m^3^, but negative effects on growth performance were avoided in the range of 5–20 kg/m^3^ when the water O_2_ concentration remained above 55%–70% saturation level (Araújo-Luna et al., 2018). Moreover, the rearing density can be increased up to 36–44 kg/m^3^ without drawback effects in growth performance when the water O_2_ level is maintained above the 100% saturation (Parma et al., 2020). Thus, as pointed out by Saraiva et al. (2022), the effects of stocking density on fish welfare are complex and would involve many interacting factors. This notion is supported at the transcriptional level by changes in the tissue-specific expression patterns with a different tissue orchestration of the stress response, according to the nature and intensity of the hypoxic and crowding stress challenge (Martos-Sitcha et al., 2019a). Thus, the liver and heart of gilthead sea bream mostly contributed to cope with a global hypoxic response, involving changes in energy sensing, antioxidant defense and tissue repair. By contrast, the skeletal muscle disclosed changes in the expression pattern of the components of the Gh/Igf system that appears more related to changes in fish density and behavioral traits rather than water O_2_ levels. In any case, wide-transcriptomic analysis in zebrafish (*Danio rerio*), Atlantic salmon and European sea bass did not highlight a conserved transcriptomic signature for a proactive behavior across fish species (Rey et al., 2021), which is perhaps indicative of the complexity and polygenic nature of the behavioral phenotype. Indeed, as discussed below, our gathered biomarker approach was able to connect proactive behavior in HD fish with locally regulated growth, while systemic growth regulation via the liver Gh/Igf system was apparently more related with a reactive coping stress style.

It is worth noting that gilthead sea bream is a schooling fish that displays social hierarchies for the use of space and competition for feed (Goldan et al., 2003; Montero et al., 2009; Arechavala-Lopez et al., 2019; Oikonomidou et al., 2019), which reinforced an enhanced physical activity in HD environments (Carbonara et al., 2019). Similarly, we found herein that measurements of body tail jerk accelerations at a high recording frequency clearly highlighted an endogenous swimming activity rhythm with a higher amplitude and adjusted-mean in the group of HD fish (Figure 3). This occurred in parallel with the rise of respiratory frequency (Figure 4A), though this in turn resulted in a slight but progressive decrease of the respiration/activity ratio from LD to HD fish (Figure 4B). Such metabolic feature would be indicative of a decreased energy partitioning for growth in a HD environment with a limited O_2_ availability, which makes sense with the observed FCR impairment in this group of fish (Table 2). Certainly, the association of better growth with a lower activity is a well-known selected pattern in livestock production (Rosenfeld et al., 2015; Sibly et al., 2015). According to this, the behavioral monitoring with the AEFishBIT accelerometer have rendered divergent patterns of energy use for growth and activity across representative European farmed fish (gilthead sea bream, European sea bass and Atlantic salmon) (Ferrer et al., 2020; Kolarevic et al., 2021; Rosell-Moll et al., 2021), but also in genetically improved gilthead sea bream for either growth or FCR (Perera et al., 2021; Calduch-Giner et al., 2023). Such genetic progress has an impact in fillet yield (Besson et al., 2022), and perhaps other productive traits affected by the establishment of different social hierarchies and interactions. In that sense, Arechavala-Lopez et al. (2020) pointed out that the time of the first gilthead sea bream response during hypoxia or risk-taking tests is shorter in HD than in LD environments, which might lead to a higher competitiveness for feed if it is not provided in a sufficient quantity and quality. At the same time, however, HD environments can serve to better synchronize the phase of locomotor and metabolic rhythms with the daily feeding time, generating an internal rhythmicity close to 24 h for a better synchronization of the individuals with the environment and their congeners (Mistlberger, 2011; Challet, 2019). This notion was fitted herein by the better cosinor adjustment of the recorded jerk accelerations in the HD fish than in the other two experimental groups (Figure 3). Feeding frequency also operates in a similar manner, and fish fed with a single meal per day showed a higher ability to concentrate the self-feeding activity around the programmed meal in comparison to those fed hourly during a 3h-window (Calduch-Giner et al., 2022). Moreover, gilthead sea bream becomes mostly arrhythmic in winter, but the use of feeding schedules with alternate days (maintaining constant the total feed intake, but with an increased supply by the feeding day) restored the typical feeding behavior of the warm season (active feeding period). The pervasiveness of biological rhythms is, thereby, adaptive in nature on a daily and/or seasonal basis, functioning as a timing reference that allows organisms to anticipate and take advantage of the diel fluctuations in their environments (Van der Zee et al., 2008). The bad thing is that the increased contact among individuals in a HD environment might lead to physical injuries (Oppedal et al., 2011; Sveen et al., 2018; Weirup et al., 2021), promoting an aggressive behavior rather than an improved individual social cohesion by the feeding time zeitgeber. In the present study, aggressive interactions among individuals are not specifically monitored, but visual observations did not reveal biting-chasing activities before-after feeding in any fish group, and more importantly, correlation analysis within the HD group highlighted a positive association between growth and skin erosion and damage (Figure 7). Therefore, it appears likely that the external body signs of welfare impairment in this group of fish (Figure 2) would be primarily due to an active feeding behavior with involuntary collisions during feed dispensation. How this non-desirable effect can be solved by more appropriate feeding schedules and housing environments is becoming an important challenge for the future development of aquaculture in a scenario of a limited space allowance for livestock production.

Plasma/serum cortisol is currently the most widely used stress biomarker in fish (Sadoul and Geffroy, 2019; Noble et al., 2020), though its reliability is limited by a number of drawbacks, including among others: i) exhaustion or adaptive HPI-axis habituation to chronic stress, ii) dramatic increase in circulating cortisol levels by sampling itself, and iii) high biological variability within and among species that hampers a clear conclusion of stress condition. This makes sense because the underlying hypothesis for the fitted low plasma cortisol levels with the increase of stocking density is that acute stress sampling can become minimized in fish coming from a HD environment (van de Nieuwegiessen et al., 2009; Santos et al., 2010; Othman et al., 2022). Furthermore, it was previously established that the regulation of gilthead sea bream plasma cortisol levels is largely dependent of the type and intensity of stressor (Bermejo-Nogales et al., 2014). It is expected, therefore, that the use of alternative water and fish scales matrix-samples gain more interest in the forthcoming years as a less invasive and reproducible cortisol assessment approach (Fanouraki et al., 2011; Weirup et al., 2021; Vercauteren et al., 2022). In the meantime, cortisol measurements should be supported by other stress indicators for an accurate assessment of fish stress/welfare status. Thus, we considered herein plasma cortisol levels as part of an integrative biomarker procedure in which the measured levels in LD fish were in the lower range of values reported for unstressed gilthead sea bream, while those from MD and HD resulted almost doubled (Table 2). The closeness found between cortisol levels of MD and HD fish can also be indicative of the different discriminant value of the assessed stress/welfare indicators. Indeed, liver and muscle gene expression patterns disclosed a different gene clustering, according to which the expression signature of MD was closer to HD in liver and to LD in muscle (Figure 5). With independence of this, the high plasma cortisol levels in HD fish were associated with a reduced growth through changes in hepatic *igf2* and *cyp7α1* expression (Figure 6), which can be considered adaptive in nature in fish facing a limited O_2_ availability that was not able to support the maximum growth of the species.

Cortisol works raising circulating glucose levels across species (Mommsen et al., 1999; Sapolsky et al., 2000), and their concurrent increase would reflect the nature and intensity of stress stimuli as well as the different stress species-specific susceptibility (Fanouraki et al., 2011; Bordin and Freire, 2021). Thus, both glucose and cortisol levels increased at a population level in a HD environment (Table 2), but the gathered biomarker analysis revealed a negative correlation between them in HD fish via the interconnections driven by different hepatic molecular transcripts (Figure 6), which might dictate a negative feedback loop that limits cortisol release promoting the use of blood glucose not only as a fast metabolic fuel, but also as a major antioxidant agent. Certainly, the rerouting of glucose into a pentose-phosphate-pathway (PPP) is a major protective mechanism to counteract acute and severe oxidative stress (Ralser et al., 2007; Stincone et al., 2015) through the production of NADPH that is required for the regeneration of reduced glutathione, a well-known antioxidant that is present in most living cells from bacteria to mammals (Hamilton et al., 2014). At the same time, however, there are several mechanisms contributing to oxidative stress during diabetes and hyperglycemia, which is indicative that exists a delicate balance between the protective and damaging effects of glucose to maintain redox homeostasis through the evolution (Cherkas et al., 2020). In the present study, the protective glucose effects in HD fish were shaped by a positive association of glucose with a number of antioxidant enzymes (*mn-sod, gpx4, prdx5*) in the liver (Figure 6) and glucose responsive proteins (*grp170, grp75*) in skeletal muscle (Figure 7), all of them already identified as highly stress-responsive genes in gilthead sea bream (Bermejo-Nogales et al., 2008; Saera-Vila et al., 2009; Calduch-Giner et al., 2010; Pérez-Sánchez et al., 2013; Malandrakis et al., 2014; Magnoni et al., 2017; Martos-Sitcha et al., 2019a; Naya-Català et al., 2021). Indeed, regardless of glucose-gene correlations, the overall trend in both liver and skeletal muscle was the upregulated expression of *grp* genes (*grp170, grp94 and grp75*) in HD fish*.* Such genes are normally overexpressed when cells are starved of glucose (Tanaka et al., 1988; Franklin et al., 2022), but the opposite is also true and the *grp75* was consistently upregulated with the increase of glycemia by crowding stress in gilthead sea bream pair-fed fish (Bermejo-Nogales et al., 2008). Likewise, when comparisons are made between gilthead sea bream and common dentex (*Dentex dentex*, a highly sensitive sparid fish to handling stress), this another sparid fish exhibited higher levels of secondary stress markers (glucose, lactate) in combination with an increased expression of pro-inflammatory cytokines and non-enzymatic antioxidant genes, such as *grp75* and metallothionein (*mt*) (Bermejo-Nogales et al., 2007). Most of these stress-responsive genes are primarily located in the mitochondria and endoplasmic reticulum, scavenging the production of reactive oxygen species (ROS) (Rahman et al., 2017; Dhamad et al., 2020). Accordingly, we found herein that the fine adjustments of metabolism in HD fish was also encompassed by the hepatic downregulation of catalytic enzyme subunits of the mitochondrial respiration chain (Complex IV; *cox1, cox2*) and the well-known lipolytic transcription factor *pparα*, which in turn resulted in the upregulated expression of a strong lipogenic marker, the *scd1a* (Table 3). This transcriptional signature is prone to promote a general depletion of oxidative metabolism, redirecting the surplus of metabolic fuels towards lipid storage as reported elsewhere during episodes of heat stress in broilers (Guo et al., 2021; Lan et al., 2022). However, this changing transcriptional signature is part of a more complex and extensive metabolic reprograming of lipid metabolism that also included a strong-down regulation of the hepatic *cyp7α1,* the first rate limiting enzyme of bile acid synthesis from cholesterol (Chiang and Ferrell, 2020). This would adjust the effective dietary fat absorption to O_2_ availability, according to the oxystatic theory that assumes that voluntary feed intake is limited by the maximal physiological capacity of O_2_ usage (Saravanan et al., 2012). Conversely, the consumption of cholesterol-rich diets can promote the shift of carbohydrate to lipid metabolism, preventing strong hypoglycemic effects following exposure to acute hypoxic stress (Miron and Tirosh, 2019). In any case, most adaptive hypoxia responses appear to be tissue-specific (Martos-Sitcha et al., 2019a), and in the absence of transcriptional changes in markers of oxidative metabolism, the skeletal muscle of HD fish disclosed a pronounced upregulation of *sirt1* and *sirt2* (Table 4). Such metabolic feature is viewed as a higher energy demanding condition that is susceptible to be epigenetically regulated in the case of *sirt1* by changes in the DNA methylation pattern of several CpG positions of a CG island close to the transcription start site (Simó-Mirabet et al., 2020). Indeed, it is well known that fasting upregulated the expression of *sirt1* in the skeletal muscle of gilthead sea bream (Simó-Mirabet et al., 2017). However, the muscle expression of *sirt2* appeared poorly responsive to nutrient deprivation, but it was upregulated in a fast growing fish strain (Simó-Mirabet et al., 2018). If this is also part of an adaptive feature to preserve muscle growth in HD fish warrants further research.

Like the confounding regulation of glucose and redox homeostasis, *Gh*-transgenic fish have a limited capacity to manage hypoxic environments efficiently (McKenzie et al., 2003; Almeida et al., 2013), though paradoxically studies in mammals indicate that circulating GH can be increased by either the increase in O_2_ requirements or the reduction in O_2_ availability (VanHelder et al., 1987). In that way, diet and exercise modulate the activity of the *Gh/Igf* axis in gilthead sea bream (Perelló-Amorós et al., 2021), and circulating *Gh* during steady states is becoming a subrogate marker of critical swimming speed (swimming activity that can be maintained theoretically indefinitely without exhaustion) (Martos-Sitcha et al., 2018). However, circulating *Gh* was lowered after acute or chronic confinement in a wide range of species, including tilapia (Aupérin et al., 1997), salmonids (Wilkinson et al., 2006) and gilthead sea bream (Saera-Vila et al., 2009). In the present study, circulating *Gh* was not measured, but the downregulated expression of hepatic *igf1* and *igf2* in HD fish highlighted a lower sensitivity of liver to the anabolic action of *Gh,* probably via post-transcriptional mechanisms because the expression of both *ghr1* and *ghr2* remained almost unaltered among all fish groups (Table 3). However, as reviewed in Pérez-Sánchez et al., 2018, and most recently stated during early development (Naya-Català et al., 2021), a large body of evidence highlighted the tissue-specific regulation of the two Ghr and Igf subtypes by nutrition and season across development in gilthead sea bream. Certainly, in contrast to that found in the liver, the muscle expression of *ghr2* was upregulated in HD fish in comparison to the other two experimental groups (Table 4), which would trigger a compensatory growth response through the enhanced expression of *igf2* as reported elsewhere in fish fed alternative feeds or semi-synthetic diets formulated to be deficient in specific nutrients (Ballester-Lozano et al., 2015; Pérez-Sánchez et al., 2018). Thus, as also pointed out before, the varying contribution of systemic (via liver Gh/Igf axis) and local growth-promoting actions on global growth are indicative of a different welfare condition and metabolic readjustment of the endocrine-growth cascade. This notion was further supported by the expression pattern of the two phylogenetically and functionally divergent clades of Igfbp1/2/4 and Igfbp3/4/6 (see Pérez-Sánchez et al., 2018), which resulted herein in an enhanced expression of hepatic *igfp1a* and muscle *igfbp3a* in HD fish (Tables 3 and 4). A common role of Igfbps among the fish lineage has not been established, but transgenic studies support a main role of Igfbp1 as a negative regulator of fish growth. Thus, *igfbp1* knockdown alleviates the hypoxic growth and development delay in zebrafish, whereas its overexpression caused growth and development retardation under normoxia (Kajimura et al., 2005). By contrast, the enhanced *igfbp3* expression has been related to growth acceleration by *Gh*-transgenesis in coho salmon (*Oncorhynchus kisutch*) (Alzaid et al., 2018). Therefore, it appears likely that all the observed transcriptional changes in the Gh/Igf axis are adaptive attempts to drive a different contribution of systemic and local growth regulatory mechanisms. According to this, our integrative biomarker survey disclosed negative correlations of hepatic Gh/Igf markers (*igf1, igf2*) with behavioral measurements of activity/respiration, whereas the opposite was found for muscle components of the Gh/Igf system (*igf2, ghr1, ghr2*), according to which this growth-regulatory transition was prone to a proactive instead of a reactive behavior. The way in which this differential tissue regulation was driven by a different threshold level of O_2_ sensors requires further warrant, though it is noteworthy that the *hif1α,* a master regulator of hypoxia-mediated responses, was apparently more sensitive to the changing crowding and hypoxic condition in muscle (*p* < 0.1) than in liver, being also positively correlated with the individual changes in growth within the HD fish population (Table 4; Figure 7).

In summary, an integrative approach depicting the way in which high stocking densities, accompanied by a decrease in O_2_ levels, drive different adaptive features in behavior, growth, antioxidant defense and lipid metabolism has been shown. Of particular relevance are the concomitant changes in behavior, antioxidant defense and growth regulatory mechanisms, which emphasizes on the importance of adaptive stress responses from the cell to the global organism level. The challenge is to translate this new knowledge into effective measures that serve to mitigate the drawback effects of a challenging and poor predictable milieu in a context of global warming, where the fish life history can largely affect the capacity of the farmed livestock to cope with most of the envisaged production threats (see Alfonso et al., 2021). According to the revisited paradigm of epigenetic nutritional/environmental regulation, this assumes the convenience of a precise and adjusted metabolic regulation over time that avoids as much as possible excessive counter-regulatory responses (Belenguer et al., 2023). Meanwhile, the present study has contributed to prescribe new and more appropriate values of welfare indicators for a better definition of a golden stocking density in a challenging environment that mimics the crowding and hypoxic stress condition of most Mediterranean farms during the summer on-growing finishing phase.

## Data Availability

The raw data supporting the conclusion of this article will be made available by the authors, without undue reservation.

## Supplementary material

SUPPLEMENTARY FIGURE S1 Welfare scoring images of **(A)** skin erosion, **(B)** caudal fin status, **(C)** pelvic fin status and **(D)** pectoral fin status. Numbers in parenthesis indicate the assigned welfare score.

SUPPLEMENTARY FIGURE S2 Gilthead sea bream physical activity synchronization by feeding at low density **(A)**, medium density **(B)** and high density **(C)**, and respiratory frequency synchronization by feeding at low density **(D)**, medium density **(E)** and high density **(F)**. AEFishBIT data (measures taken every 15 min along 2 consecutive days) of representative individuals (*n* = 8) is shown as a continuous dotted line in each panel. Best-fit curves (red sinusoidal line) derived from the cosinor analysis of physical activity and respiratory frequency are represented in the HD group. Values of mesor (M), amplitude (A), acrophase (φ) and *p*-value (*P*) of best-fit curves are shown for each density. Gray shaded areas represent dark phases. Arrow in vertical dotted line indicates the feeding time. Different letters represent significant (*P* < 0.05, ANOVA, Holm-Sidak test) differences between density groups.

SUPPLEMENTARY FIGURE S3 Validation plots of the PLS-DA models in Figures 5A, B **(A,B)** consisting in 500 random permutations each.

SUPPLEMENTARY TABLE S3 Pearson correlation matrix among outcomes on growth performance, skin damage, behavior, plasma stress markers and hepatic gene expression in HD fish. Upper values are the correlation coefficients and lower values are the *p*-values.

SUPPLEMENTARY TABLE S4 Pearson correlation matrix among outcomes on growth performance, skin damage, behavior, plasma stress markers and muscle gene expression in HD fish. Upper values are the correlation coefficients and lower values are the *p*-values.
