# Supplementary figures and images for: Understanding how high stocking densities and concurrent limited oxygen availability drive social cohesion and adaptive features in regulatory growth, antioxidant defense and lipid metabolism in farmed gilthead sea bream (Sparus aurata)
Source: Front Physiol. 2023 Oct 4;14:1272267. doi: 10.3389/fphys.2023.1272267 (PMC10586056; doi:10.3389/fphys.2023.1272267)

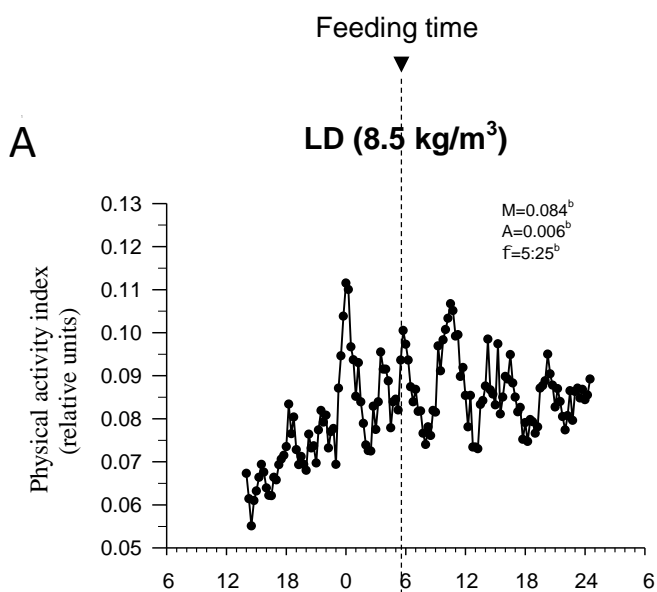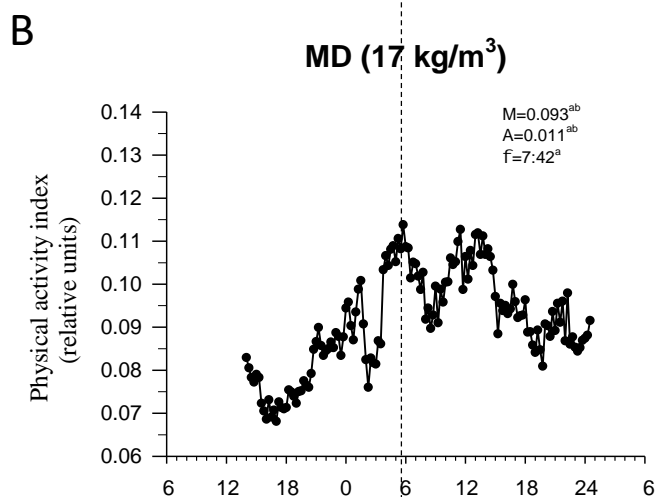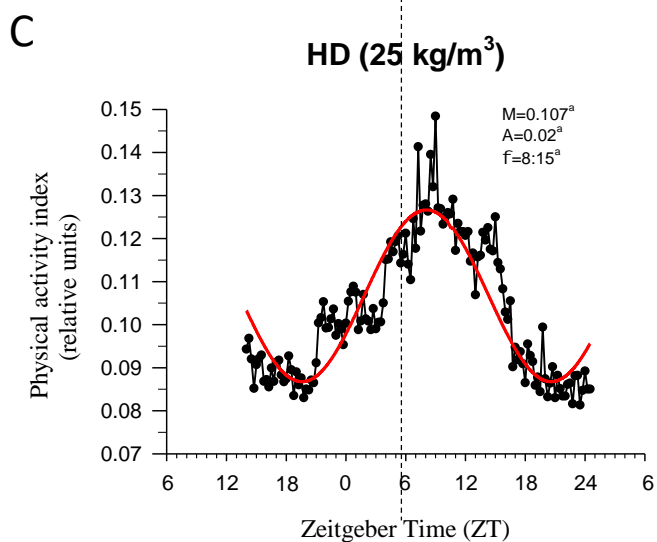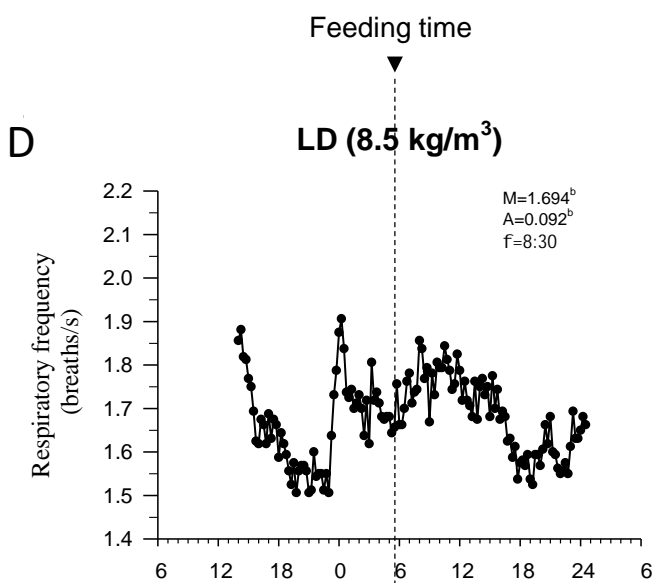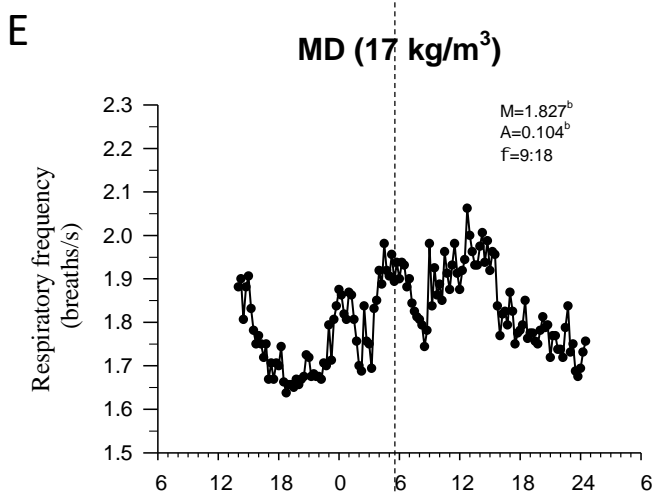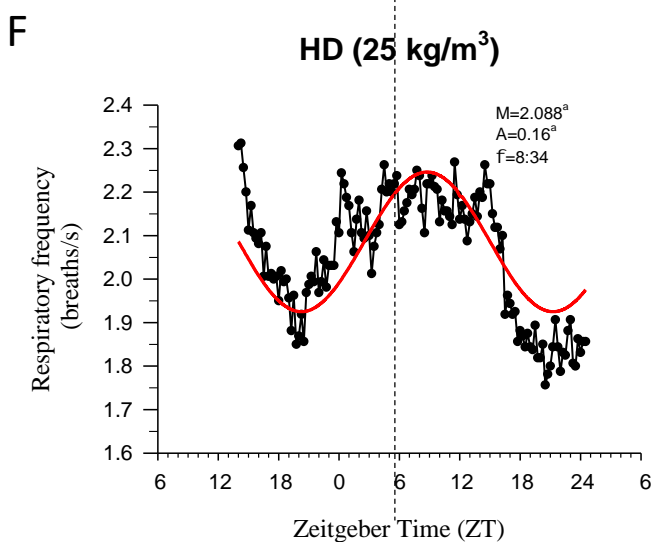

Supplement: Supplementary file 1 [file DataSheet2.PDF]

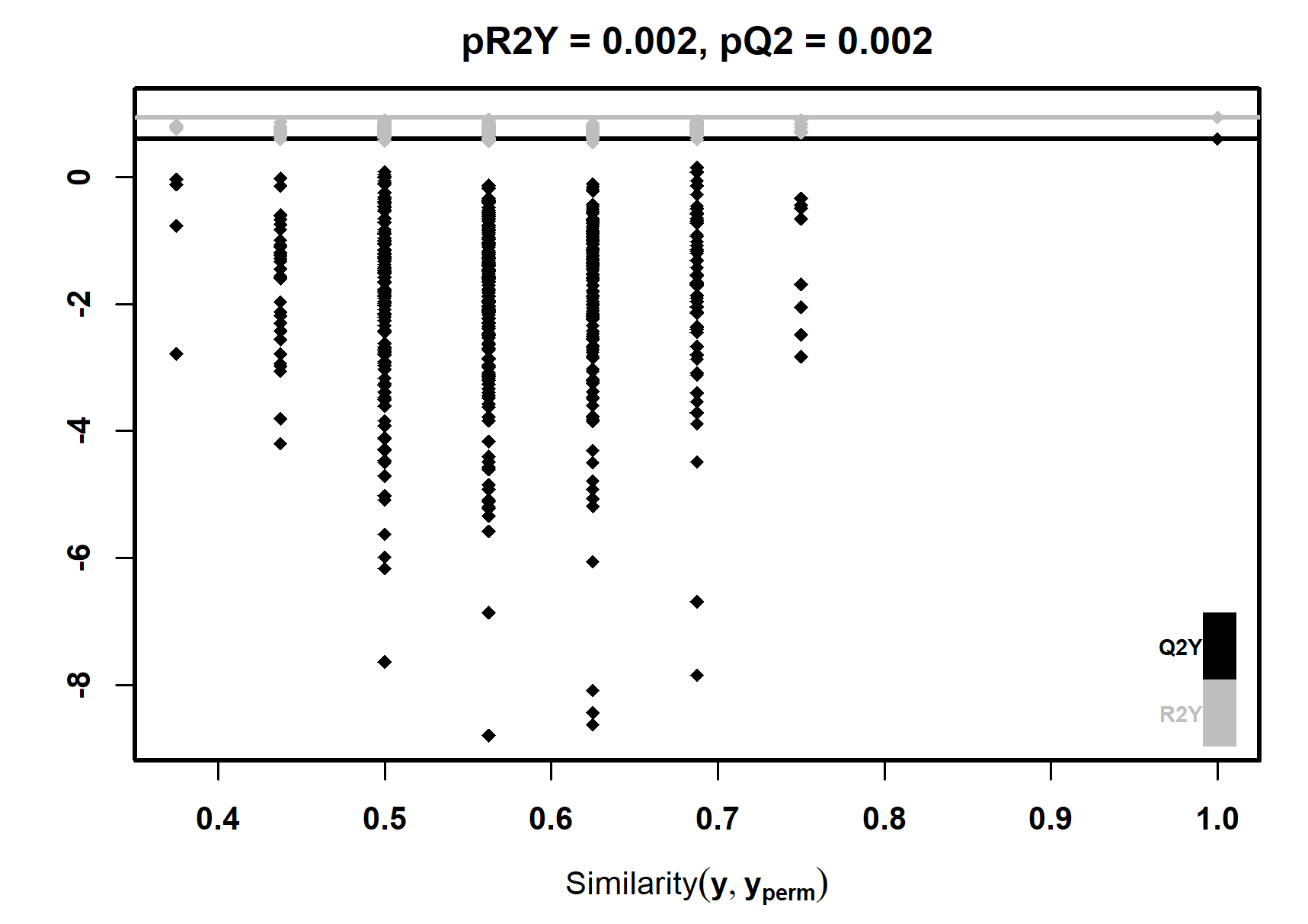

Supplement: Supplementary file 4 [file Image2.TIF]

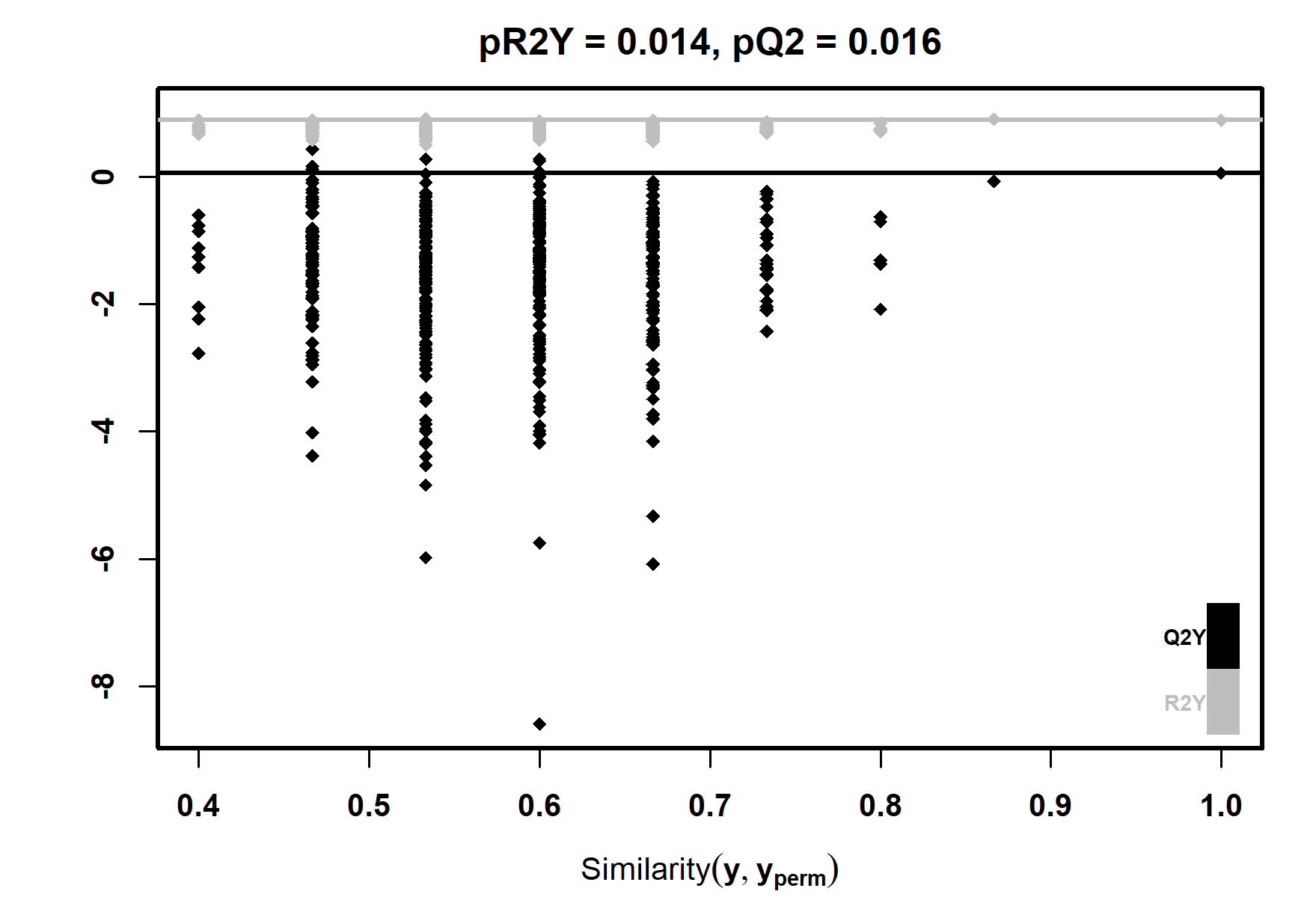

Supplement: Supplementary file 5 [file Image1.TIF]

A

## Skin erosion

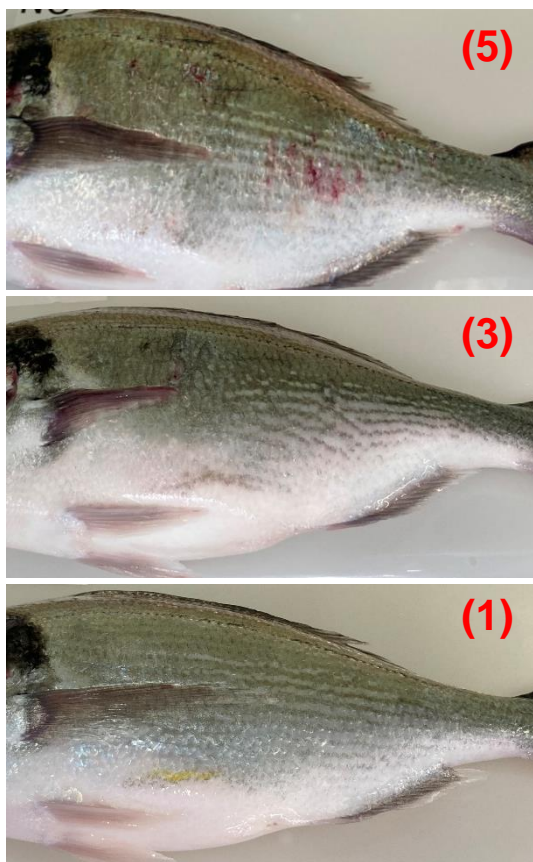

B

## Caudal fin status

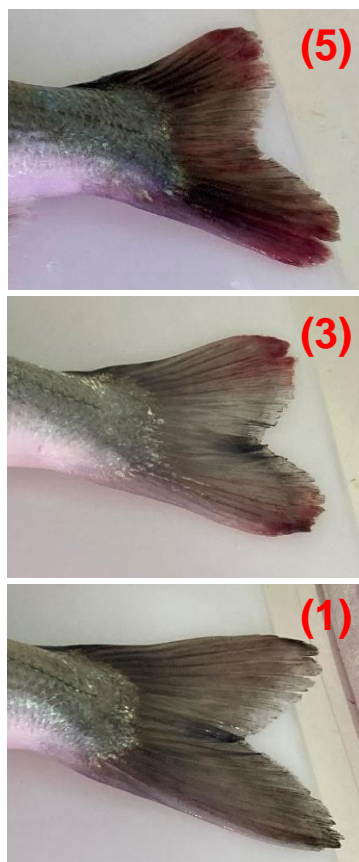

C

## Pelvic fin status

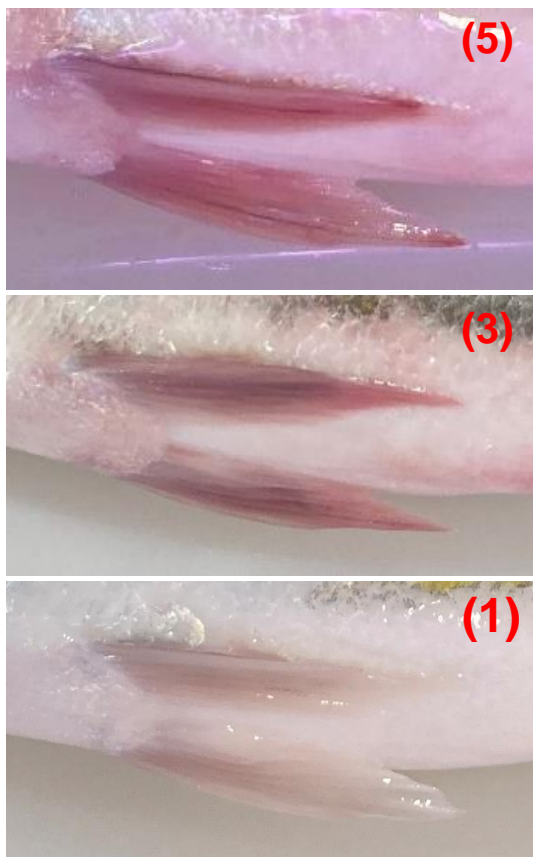

D

## Pectoral fin status

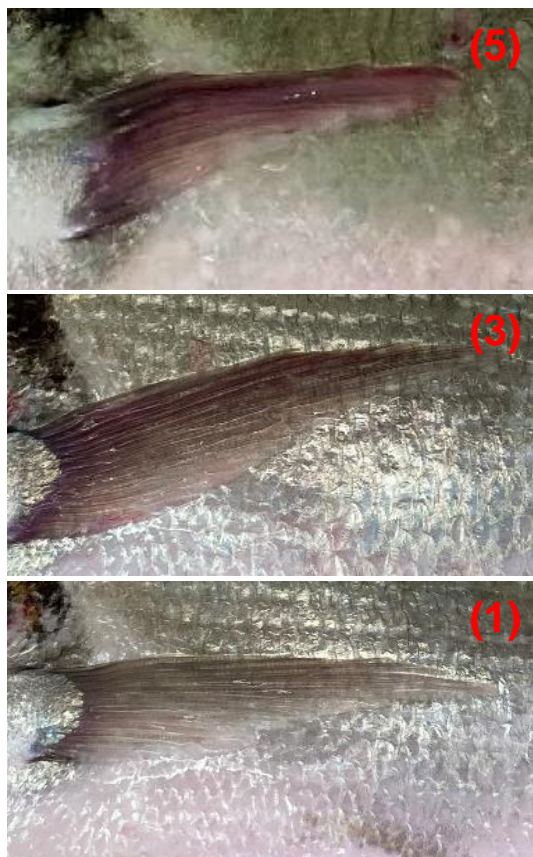

Supplement: Supplementary file 7 [file DataSheet1.PDF]
