# Supplementary material for: Understanding how high stocking densities and concurrent limited oxygen availability drive social cohesion and adaptive features in regulatory growth, antioxidant defense and lipid metabolism in farmed gilthead sea bream (Sparus aurata)
Source: Front Physiol. 2023 Oct 4;14:1272267. doi: 10.3389/fphys.2023.1272267 (PMC10586056; doi:10.3389/fphys.2023.1272267)
Supplement: Supplementary file 3 [file DataSheet4.PDF]

Supplementary Table 2. Primers for qPCR amplification of white skeletal muscle genes.

Gene	Symbol	GenBank	Primer
Growth hormone receptor-type 1	*ghr1*	AF438176	F: ACC TGT CAG CCA CCA CAT GA<br>R: TCG TGC AGA TCT GGG TCG TA
Growth hormone receptor-type 2	*ghr2*	AY573601	F: GAG TGA ACC CGG CCT GAC AG<br>R: GCG GTG GTA TCT GAT TCA TGG T
Insulin-like growth factor 1	*igf1*	AY996779	F: TGT CTA GCG CTC TTT CCT TTC A<br>R: AGA GGG TGT GGC TAC AGG AGA TAC
Insulin-like growth factor 2	*igf2*	AY996778	F: TGG GAT CGT AGA GGA GTG TTG T<br>R: CTG TAG AGA GGT GGC CGA CA
Insulin-like growth factor binding protein 3a	*igfbp3a*	MH577191	F: ACA GGC GTG TGG AGT GTA<br>R: TGG TGC TGG CAG GTC AAG
Insulin-like growth factor binding protein 3b	*igfbp3b*	MH577192	F: GCC AGA TTA TGG TCC CTG TCG GAG AGA G<br>R: GTC TGT AAT CTT GAG GCT GCT GAG GAT GCT
Insulin-like growth factor binding protein 5a	*igfbp5a*	MH577193	F: GAA TCT CAC GAT GAC GCC AT<br>R: TGC TGA TGT GGT CTC TTC C
Insulin-like growth factor binding protein 5b	*igfbp5b*	MH577194	F: GCA AGC AGT GTA AGC CAT CTC<br>R: TGA ACG CCG TAC TTG TCC A
Insulin-like growth factor binding protein 6a	*igfbp6a*	MH577195	F: CGA CCC GAA TCA CGA CAT ATA CAT<br>R: ACT TGC CAC GCC GCT TAC
Insulin-like growth factor binding protein 6b	*igfbp6b*	MH577196	F: GAT TGC TCA CTG CGG ATC<br>R: GGA GGG ACA GAC CTT GAA
Myoblast determination protein 1	*myod1*	AF478568	F: ATG GAG CTG TCG GAT ATC TCT TTC<br>R: GAA GCA GGG GTC ATC GTA GAA ATC
Myogenic determination protein 2	*myod2*	AF478569	F: CCA ACT GCT CTG ATG GCA TGA TGG ATT TC<br>R: GAC CGT TTG CTT CTC CTG GAC TCG TAT G
Myogenic factor 5	*myf5*	JN034420	F: GCA TGG TTG ACA GCA ACA GTC CAG TGT<br>R: TGT CTT ATC GCC CAA AGT GTC GTT CTT CAT
Myogenic factor 6	*myf6/herculin*	JN034421	F: GCA GCA ATG ACA AAC CAG AGA GAC GGA ACA<br>R: GAG GCT GGA GGA CGC CGA AGA TTC A
Myostatin/Growth differentiation factor 8	*mstn/gdf8*	AF258448	F: AAG AGC AGA TCA TCT ACG GCA AGA TCC<br>R: TCA AGA GCA TCC ACA ACG GTC TAC CA
Myocyte-specific enhancer factor 2a	*mef2a*	KM522777	F: ATG GAC GAG AGG AAC AGG CAG GTT A<br>R: GGC TAT CTC ACA GTC ACA TAG TAC GCT CAG
Myocyte-specific enhancer factor 2c	*mef2c*	KM522778	F: TAG CAA CTC CCA CTC TAC CAG GAC AAG<br>R: GGA ATA CTC GGC ACC ATA AGA AGT CG
Follistatin	*fst*	AY544167	F: GGA CCA GAC AAA CAA CGC ATA TTG<br>R: CAT AGA TGA TCC CGT CGT TTC CAC
Interleukin 1*β*	*il1β*	AJ419178	F: GCG ACC TAC CTG CCA CCT ACA CC<br>R: TCG TCC ACC GCC TCC AGA TGC
Interleukin 6	*il6*	EU244588	F: TCT TGA AGG TGG TGC TGG AAG TG<br>R: AAG GAC AAT CTG CTG GAA GTG AGG
Interleukin 8	*il8*	JX976619	F: CAG CAG AGT CTT CAT CGT CAC TAT TG<br>R: AGG CTC GCT TCA CTG ATG G
Interleukin 10	*il10*	JX976621	F: AAC ATC CTG GGC TTC TAT CTG<br>R: GTG TCC TCC GTC TCA TCT G

Interleukin 12 subunit *β*	*il12β*	JX976624	F: ATT CCC TGT GTG GTG GCT GCT<br>R: GCT GGC ATC CTG GCA CTG AAT
Hypoxia inducible factor 1*α*	*hif1α*	JQ308830	F: CAG ATG AGC CTC TAA CTT GTG GAC<br>R: TTA GCA AGA ATG GTG GCA AGA TGA G
Proliferator-activated receptor γ coactivator 1*α*	*pgc1α*	JX975264	F: CGT GGG ACA GGT GTA ACC AGG ACT C<br>R: ACC AAC CAA GGC AGC ACA CTC TAA TTC T
Proliferator-activated receptor γ coactivator 1*β*	*pgc1β*	JX975265	F: TCA GAG GAA GAG GCG GAT<br>R: GAC ACA GGT GGA GGA TGG
Carnitine palmitoyltransferase 1a	*cpt1a*	JQ308822	F: GTG CCT TCG TTC GTT CCA TGA TC<br>R: TGA TGC TTA TCT GCT GCC TGT TTG
Citrate synthase	*cs*	JX975229	F: TCC AGG AGG TGA CGA GCC<br>R: GTG ACC AGC AGC CAG AAG AG
NADH-ubiquinone oxidoreductase chain 2	*nd2*	KC217558	F: TAG GTT GAA TGA CCA TCG TA<br>R: GGC TAA GGA GTT GAG GTT
NADH-ubiquinone oxidoreductase chain 5	*nd5*	KC217559	F: CCT AAA CGC CTG AGC CCT GG<br>R: GCT GTA AAC GAG GTG GCT AGA AGG
Cytochrome c oxidase subunit 1	*cox1*	KC217652	F: GTC CTA CTT CTT CTG TCC CTT CCT GTT CT<br>R: AGG TTT CGG TCT GTA AGG AGC ATT GTA ATC
Cytochrome c oxidase subunit 2	*cox2*	KC217653	F: ACT GCC TAC ACA GGA CCT TGC C<br>R: GTC TGC TTC CAG GAG ACG GAA TTG T
Uncoupling protein 3	*ucp3*	EU555336	F: AGG TGC GAC TGG CTG ACG<br>R: TTC GGC ATA CAA CCT CTC CAA AG
Sirtuin1	*sirt1*	KF018666	F: GGT TCC TAC AGT TTC ATC CAG CAG CAC ATC<br>R: CCT CAG AAT GGT CCT CGG ATC GGT CTC
Sirtuin2	*sirt2*	KF018667	F: GAA CAA TCC GAC GAC AGC AGT GAA G<br>R: AGG TTA CGC AGG AAG TCC ATC TCT
Catalase	*cat*	JQ308823	F: TGG TCG AGA ACT TGA AGG CTG TC<br>R: AGG ACG CAG AAA TGG CAG AGG
Glutathione peroxidase 4	*gpx4*	AM977818	F: TGC GTC TGA TAG GGT CCA CTG TC<br>R: GTC TGC CAG TCC TCT GTC GG
Glutathione reductase	*gr*	AJ937873	F: TGT TCA GCC ACC CAC CCA TCG G<br>R: GCG TGA TAC ATC GGA GTG AAT GAA GTC TTG
Peroxiredoxin 3	*prdx3*	GQ252681	F: ATC AAC ACC CCA CGC AAG ACT G<br>R: ACC GTT TGG ATC AAT GAG GAA CAG ACC
Peroxiredoxin 5	*prdx5*	GQ252683	F: GAG CAC GGA ACA GAT GGC AAG G<br>R: TCC ACA TTG ATC TTC TTC ACG ACT CC
Superoxide dismutase [Mn]	*mn-sod / sod2*	JQ308833	F: CCT GAC CTG ACC TAC GAC TAT GG<br>R: AGT GCC TCC TGA TAT TTC TCC TCT G
Glucose-regulated protein 170 kDa	*grp170*	JQ308821	F: CAG AGG AGG CAG ACA GCA AGA C<br>R: TTC TCA GAC TCA GCA TTT CCA GAT TTC
Glucose-regulated protein 94 kDa	*grp94*	JQ308820	F: AAG GCA CAG GCT TAC CAG ACA G<br>R: CTT CAG CAT CAT CGC CGA CTT TC
Glucose-regulated protein 75 kDa	*grp75*	DQ524993	F: TCC GGT GTG GAT CTG ACC AAA GAC<br>R: TGT TTA GGC CCA GAA GCA TCC ATG
Beta-actin	*actb*	KY388508	F: TCC TGC GGA ATC CAT GAG A<br>R: GAC GTC GCA CTT CAT GAT GCT
