# Supplementary material for: Understanding how high stocking densities and concurrent limited oxygen availability drive social cohesion and adaptive features in regulatory growth, antioxidant defense and lipid metabolism in farmed gilthead sea bream (Sparus aurata)
Source: Front Physiol. 2023 Oct 4;14:1272267. doi: 10.3389/fphys.2023.1272267 (PMC10586056; doi:10.3389/fphys.2023.1272267)
Supplement: Supplementary file 6 [file DataSheet3.PDF]

Supplementary Table 1. Primers for qPCR amplification of hepatic genes.

Gene	Symbol	GenBank	Primer
Growth hormone receptor-type 1	*ghr1*	AF438176	F: ACC TGT CAG CCA CCA CAT GA<br>R: TCG TGC AGA TCT GGG TCG TA
Growth hormone receptor-type 2	*ghr2*	AY573601	F: GAG TGA ACC CGG CCT GAC AG<br>R: GCG GTG GTA TCT GAT TCA TGG T
Insulin-like growth factor 1	*igf1*	AY996779	F: TGT CTA GCG CTC TTT CCT TTC A<br>R: AGA GGG TGT GGC TAC AGG AGA TAC
Insulin-like growth factor 2	*igf2*	AY996778	F: TGG GAT CGT AGA GGA GTG TTG T<br>R: CTG TAG AGA GGT GGC CGA CA
Insulin-like growth factor binding protein 1a	*igfbp1a*	KM522771	F: ACA AAC CAA AAC AGT GCG AGT CCT C<br>R: CCG TTC CAA GAG TTC ACA CAC CAG
Insulin-like growth factor binding protein 1b	*igfbp1b*	MH577189	F: GCC AAA CAG TGT GAG TCA TC<br>R: ATC TTC TTC CCG TTC CAG G
Insulin-like growth factor binding protein 2a	*igfbp2a*	MH577190	F: CCA GCA AAG AGA CCA CCT<br>R: TCT TCA TCT CCT GCC TGT G
Insulin-like growth factor binding protein 2b	*igfbp2b*	AF377998	F: AGC GAT GTG TCC TGA GAT AGT GAG<br>R: GCA CCG TGG CGT GTA GAC C
Insulin-like growth factor binding protein 4	*igfbp4*	KM658998	F: GGC ATC AAA CAC CCG CAC AC<br>R: ATC CAC GCA CCA GCA CTT CC
Elongation of very long chain fatty acids 1	*elovl1*	JX975700	F: CTT CCT ACA CAT CTT CCA CCA CTC<br>R: CCA TTC CAC CAG GAG CAA AGG
Elongation of very long chain fatty acids 4	*elovl4*	JX975701	F: CGG TGG CAA TCA TCT TCC<br>R: TCA ACT GGC TGT CTG TGT
Elongation of very long chain fatty acids 5	*elovl5*	AY660879	F: CCT CCT GGT GCT CT ACA AT<br>R: GTG AGT GTC CTG GCA GTA
Elongation of very long chain fatty acids 6	*elovl6*	JX975702	F: GTG CTG CTC TAC TCC TGG TA<br>R: ACG GCA TGG ACC AAG TAG T
Fatty acid desaturase 2	*fads2*	AY055749	F: GCA GGC GGA GAG CGA CGG TCT GTT CC<br>R: AGC AGG ATG TGA CCC AGG TGG AGG CAG AAG
Stearoyl-CoA desaturase 1a	*scd1a*	JQ277703	F: CGG AGG CGG AGG CGT TGG AGA AGA AG<br>R: AGG GAG ACG GCG TAC AGG GCA CCT ATA TG
Stearoyl-CoA desaturase 1b	*scd1b*	JQ277704	F: GCT CAA TCT CAC CAC CGC CTT CAT AG<br>R: GCT GCC GTC GCC CGT TCT CTG
Hepatic lipase	*hl*	EU254479	F: TTG TAG AAG GTG AGG AAA ACT G<br>R: GCT CTC CAT CAG ACC ATC C
Lipoprotein lipase	*lpl*	AY495672	F: CGT TGC CAA GTT TGT GAC CTG<br>R: AGG GTG TTC TGG TTG TCT GC
Adipose triglyceride lipase	*atgl*	JX975711	F: GTG CTT CAG TCC TGG ATG TCT TC<br>R: AGC CTT GCA GGT CCA TGT TGA
85kDa calcium-independent phospholipase A2	*pla2g6*	JX975708	F: CGC CAA GGA ACT CGG AAA GAT GCT<br>R: ACC GCA CAG CCA TCA GAG TCT
Cholesterol 7-alpha-monooxygenase	*cyp7a1*	KX122017	F: CCC TGC TAT TAA AGT CCC ACC TCT<br>R: ATC GTA GGT AGG CTG GAG GAT TC
Peroxisome proliferator-activated receptor α	*pparα*	AY590299	F: TCT CTT CAG CCC ACC ATC CC<br>R: ATC CCA GCG TGT CGT CTC C

Peroxisomeproliferator-activated receptor γ	*pparγ*	AY590304	F: CGC CGT GGA CCT GTC AGA GC<br>R: GGA ATG GAT GGA GGA GGA GGA GAT GG
Hypoxia inducible factor 1*α*	*hif1α*	JQ308830	F: CAG ATG AGC CTC TAA CTT GTG GAC<br>R: TTA GCA AGA ATG GTG GCA AGA TGA G
Proliferator-activated receptor γ coactivator 1*α*	*pgc1α*	JX975264	F: CGT GGG ACA GGT GTA ACC AGG ACT C<br>R: ACC AAC CAA GGC AGC ACA CTC TAA TTC T
Carnitine palmitoyltransferase 1a	*cpt1a*	JQ308822	F: GTG CCT TCG TTC GTT CCA TGA TC<br>R: TGA TGC TTA TCT GCT GCC TGT TTG
Fatty acid binding protein, heart	*hfabp*	JQ308834	F: CTG GGT GTG GGC TTC GCT AC<br>R: CTC TGT GTT CTT GAT GGT GCT CTG
Citrate synthase	*cs*	JX975229	F: TCC AGG AGG TGA CGA GCC<br>R: GTG ACC AGC AGC CAG AAG AG
NADH-ubiquinone oxidoreductase chain 2	*nd2*	KC217558	F: TAG GTT GAA TGA CCA TCG TA<br>R: GGC TAA GGA GTT GAG GTT
NADH-ubiquinone oxidoreductase chain 5	*nd5*	KC217559	F: CCT AAA CGC CTG AGC CCT GG<br>R: GCT GTA AAC GAG GTG GCT AGA AGG
Cytochrome c oxidase subunit 1	*cox1*	KC217652	F: GTC CTA CTT CTT CTG TCC CTT CCT GTT CT<br>R: AGG TTT CGG TCT GTA AGG AGC ATT GTA ATC
Cytochrome c oxidase subunit 2	*cox2*	KC217653	F: ACT GCC TAC ACA GGA CCT TGC C<br>R: GTC TGC TTC CAG GAG ACG GAA TTG T
Uncoupling protein 1	*ucp1*	FJ710211	F: GCA CAC TAC CCA ACA TCA CAA G<br>R: CGC CGA ACG CAG AAA CAA AG
Sirtuin1	*sirt1*	KF018666	F: GGT TCC TAC AGT TTC ATC CAG CAG CAC ATC<br>R: CCT CAG AAT GGT CCT CGG ATC GGT CTC
Sirtuin2	*sirt2*	KF018667	F: GAA CAA TCC GAC GAC AGC AGT GAA G<br>R: AGG TTA CGC AGG AAG TCC ATC TCT
Glutathione peroxidase 1	*gpx1*	DQ524992	F: GAA GGT GGA TGT GAA TGG AAA AGA TG<br>R: CTG ACG GGA CTC CAA ATG ATG G
Glutathione peroxidase 4	*gpx4*	AM977818	F: TGC GTC TGA TAG GGT CCA CTG TC<br>R: GTC TGC CAG TCC TCT GTC GG
Peroxiredoxin 3	*prdx3*	GQ252681	F: ATC AAC ACC CCA CGC AAG ACT G<br>R: ACC GTT TGG ATC AAT GAG GAA CAG ACC
Peroxiredoxin 5	*prdx5*	GQ252683	F: GAG CAC GGA ACA GAT GGC AAG G<br>R: TCC ACA TTG ATC TTC TTC ACG ACT CC
Superoxide dismutase [Cu-Zn]	*cu-zn-sod / sod1*	JQ308832	F: TCA CGG ACA AGA TGC TCA CTC TC<br>R: GGT TCT GCC AAT GAT GGA CAA GG
Superoxide dismutase [Mn]	*mn-sod / sod2*	JQ308833	F: CCT GAC CTG ACC TAC GAC TAT GG<br>R: AGT GCC TCC TGA TAT TTC TCC TCT G
Glucose-regulated protein 170 kDa	*grp170*	JQ308821	F: CAG AGG AGG CAG ACA GCA AGA C<br>R: TTC TCA GAC TCA GCA TTT CCA GAT TTC
Glucose-regulated protein 94 kDa	*grp94*	JQ308820	F: AAG GCA CAG GCT TAC CAG ACA G<br>R: CTT CAG CAT CAT CGC CGA CTT TC
Glucose-regulated protein 75 kDa	*grp75*	DQ524993	F: TCC GGT GTG GAT CTG ACC AAA GAC<br>R: TGT TTA GGC CCA GAA GCA TCC ATG
Beta-actin	*actb*	KY388508	F: TCC TGC GGA ATC CAT GAG A<br>R: GAC GTC GCA CTT CAT GAT GCT
